# Effects of Lean Interventions Supported by Digital Technologies on Healthcare Services: A Systematic Review

**DOI:** 10.3390/ijerph19159018

**Published:** 2022-07-25

**Authors:** Diego Tlapa, Guilherme Tortorella, Flavio Fogliatto, Maneesh Kumar, Alejandro Mac Cawley, Roberto Vassolo, Luis Enberg, Yolanda Baez-Lopez

**Affiliations:** 1 Facultad de Ingeniería, Arquitectura y Diseño, Universidad Autonoma de Baja California, Ensenada 23080, Mexico; yolanda@uabc.edu.mx; 2 Department of Mechanical Engineering, The University of Melbourne, Melbourne, VIC 3010, Australia; guilherme.tortorella@unimelb.edu.au; 3 Industrial Engineering Department, Universidade Federal do Rio Grande do Sul, Porto Alegre 90010-150, Brazil; ffogliatto@gmail.com; 4 Cardiff Business School, Cardiff University, Cardiff CF5 2YB, UK; kumarm8@cardiff.ac.uk; 5 Departamento de Ingeniería Industrial y de Sistemas, Pontificia Universidad Católica de Chile, Santiago 8331150, Chile; amac@ing.puc.cl (A.M.C.); luisenberg@gmail.com (L.E.); 6 IAE Business School, Universidad Austral, Buenos Aires B1630FHB, Argentina; rvassolo@iae.edu.ar

**Keywords:** Healthcare 4.0, lean healthcare, automation, simulation, process improvement

## Abstract

Despite the increasing utilization of lean practices and digital technologies (DTs) related to Industry 4.0, the impact of such dual interventions on healthcare services remains unclear. This study aims to assess the effects of those interventions and provide a comprehensive understanding of their dynamics in healthcare settings. The methodology comprised a systematic review following the PRISMA guidelines, searching for lean interventions supported by DTs. Previous studies reporting outcomes related to patient health, patient flow, quality of care, and efficiency were included. Results show that most of the improvement interventions relied on lean methodology followed by lean combined with Six Sigma. The main supporting technologies were simulation and automation, while emergency departments and laboratories were the main settings. Most interventions focus on patient flow outcomes, reporting positive effects on outcomes related to access to service and utilization of services, including reductions in turnaround time, length of stay, waiting time, and turnover time. Notably, we found scarce outcomes regarding patient health, staff wellbeing, resource use, and savings. This paper, the first to investigate the dual intervention of DTs with lean or lean–Six Sigma in healthcare, summarizes the technical and organizational challenges associated with similar interventions, encourages further research, and promotes practical applications.

## 1. Introduction

Improving healthcare quality and efficiency is a recurring challenge faced by healthcare services. Internal inefficiencies, such as poor patient flow and inadequate resource utilization [1], may contribute to overcrowding and delays in care [2], affecting patient and staff satisfaction, patient safety, and the overall quality of care [3,4]. To improve healthcare quality and efficiency, healthcare providers have searched outside their sector for guidance [5]. Several methodologies and techniques have been tested, most notably lean healthcare (LH) and Six Sigma. LH has been a recurrent intervention for increasing efficiency by reducing non-value-added activities, whereas Six Sigma is a methodology focused on reducing variation in processes or services [6,7,8,9,10,11,12,13,14].

Improving efficiency and quality of care can be further enhanced by applying digital technologies (DTs) offered by Industry 4.0. From replacing dry boards with computerized patient-tracking systems in emergency departments (EDs) [15] to replacing human observers with wireless tags (real-time locating systems) for determining the location of patients and staff [16], DTs benefit healthcare services. Healthcare 4.0 adapts applications and principles from Industry 4.0 to healthcare settings, allowing traceability, real-time visibility [17], and care customization to professionals and patients [18]. DTs of Healthcare 4.0 are varied, including telemedicine [19], machine learning [20], deep learning [21], big data [22], automation [23], simulation [24], and blockchain [25], among others. Different approaches to organizing DTs have been proposed, e.g., based on the extent of their patient-centered integration and caregiver interaction [26], their roles and applicability within the hospital [27], the solutions provided by each technology [28], the link between DTs and service processes [29], or the main beneficiary [30]. The diversity of approaches indicates that consensus is yet to be achieved on the bundles of DTs best suited for each purpose [31]. In this regard, we have not identified the best bundles of DTs in the context of LH interventions, which suggests a research gap to be bridged and gives rise to the first research question:

RQ1. Which DTs support LH interventions?

LH interventions supported by DTs vary in purpose. They are reported in different healthcare settings, e.g., using fuzzy logic to assess the leanness of a supply chain in healthcare [32], transforming an emergency department workflow combining LH, machine learning, and simulation [20], or optimizing antibiotic administration through LH and automation [33]. Despite the increasing popularity and adoption of LH and DTs, there is concern regarding implementation failures since not all organizations have experienced the same level of success. For example, a case study by Moo-Young et al. [34] reported a decrease in patients’ length of stay but no significant improvements in discharge order time and response time. Similar cases with mixed results are found in the literature [33,35]. Different studies have addressed LH’s barriers, benefits, and evolution [36,37,38,39,40,41,42,43,44,45,46,47,48]. Likewise, authors have identified factors that might affect the implementation of DTs in healthcare, including contingency factors [49] and enablers [50], whereas others aimed at tracking the progress, effects, barriers, limitations, and context of DT implementations [18,30,31,51,52,53,54]. As a dual intervention, LH and DTs have been studied mainly in manufacturing companies [55,56,57,58]; however, studies on DTs supporting improvement interventions in healthcare such as LH and Six Sigma and the effects of such interventions are not available. Based on these arguments, a second research question emerges:

RQ2. What are the effects of LH interventions supported by DTs on healthcare services?

To address the research questions above, we conducted a systematic review to organize, classify, and summarize the literature on LH interventions supported by DTs. Despite the number of studies reporting lean interventions, we did not find published studies focusing on the dynamics of lean interventions in healthcare supported by digital technologies associated with Industry 4.0. This is the first paper that investigates the effects of such interventions on healthcare services and determines the technologies utilized, the types of settings, and the challenges involved in the dual interventions. Hence, we believe our results to be highly valuable for practitioners and researchers.

## 2. Theoretical Framework

### 2.1. Healthcare 4.0 Digital Technologies

Driven by the application of DTs offered by Industry 4.0, hospitals have accelerated their digitalization across all settings and processes. The digitalization of healthcare is known by various names, including smart health [59], e-health [60], Health 4.0 [30,61], Care 4.0 [62], and Healthcare 4.0 [63,64,65], among others. Such digital transformation has provided an increasingly supportive environment to improve efficiency and the quality of care. In addition, the exponential growth of healthcare data is another major issue in current healthcare information systems; thus, different DTs have been used to perform analytics, including descriptive, predictive, and prescriptive analytics, by using big data [66], e.g., a big data application to provide information on the entire customer experience in healthcare [22]. Similarly, machine learning has been used to support the prediction of patient characteristics, resource needs, treatment outcomes, and re-admission patterns [20].

In addition, due to the inherent complexity of healthcare systems, different simulation modeling techniques have been used for a wide range of applications in healthcare [24,67]. Along with simulation, automation has also been increasingly used, e.g., an automated dispensing cabinet used to administrate in a timely manner antibiotics for patients with severe sepsis [33] or automated instrumentation to increase the effectiveness of urinalysis tests [68]. Related to automation, robot systems have been used for ultraviolet disinfection in hospital rooms to prevent environmental transmission of pathogens, thus improving room turnover efficiency [69]. Additional DTs include an electronic patient-tracking system simulator that combines a realistic model of ED with patient events, helping to assess the situation awareness and workload of the ED staff [15]; sensors and digital counters for the reduction of door movement during surgery [70]; and telemedicine, a technology that has been used for several years but that experienced a significant increase due to the COVID-19 pandemic [19]. More applications of Healthcare 4.0 range from customization of implants and digital hospitals, to virtual reality and treatment monitoring [71].

### 2.2. Lean Interventions

Lean is derived from the Toyota production system (TPS), a system originally used to increase efficiency in manufacturing companies [72]. More recently, TPS has also been identified as an effective strategy to lower costs and improve outcomes in the healthcare sector [73]. The prevalence of LH permeates several healthcare services and specialties, e.g., intensive care units (ICUs) [6], cardiology [7], surgery [8], colonoscopy [74], pathology [9], radiology [75], mental health [76], eye hospitals [10], and clinical laboratories [77]. LH starts by reviewing a healthcare process to determine what is of value to the patient, i.e., activities that enhance healthcare quality and promote patient well-being towards a better outcome [78]. Correspondingly, LH helps identify waste, i.e., anything other than the minimum amount of equipment, space, or staff time essential to add value to a product or service [79]. As a result, LH classifies activities into value-added (VA) or non-value-added (NVA) [80]. Value-added activities contribute directly to patient needs, whereas non-value-added activities take unnecessary time, space, or resources [80,81].

### 2.3. Main Outcomes

Health system interventions might include several outcomes to measure their effectiveness. Commonly reported outcomes in LH or DT interventions include those suggested by the EPOC group, whose categories are clustered into main and secondary outcomes [82]. Within the former, patient outcomes is a category that includes the 30-day mortality rate [6,33,83,84] and the readmission or revisits rate [6,83]. Outcomes related to the utilization of services include the length of stay (LOS) [85,86] and discharge order time [87,88]. Access to service outcomes include patient waiting time [78,89], boarding time [3,87], and the number of patients who left without being seen (LWBS) [20]. Regarding resource use, turnaround time [68,90], turnover time [69], and on-time starts [91,92,93] are common outcomes in the literature. Among the secondary outcomes, patient, staff, and stakeholder satisfaction are indicators commonly measured [82].

As previously reported [94], patient flow, i.e., the movement of patients through care settings [95], is a common focus of attention when it comes to efficiency for both LH and DT interventions. However, patient flow might be impacted by different outcomes, including the waiting time of a patient to be seen by a healthcare professional, the LOS once the patient has been seen, the turnover or turnaround time, and even the boarding time. Our research aims to review the effect of LH and DT interventions on healthcare services, including the patient flow and the outcomes suggested by the EPOC group [82].

## 3. Materials and Methods

**Figure 1:**
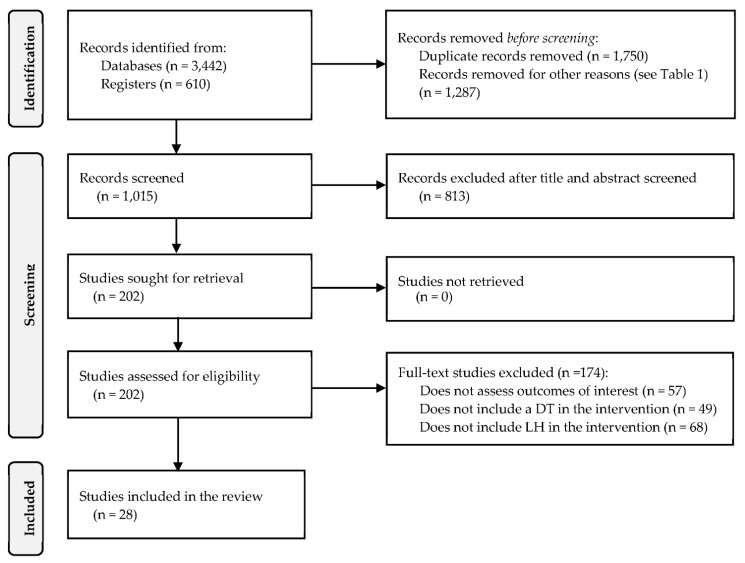
PRISMA flow chart.

A systematic review was performed based on the PRISMA guidelines [96,97,98] and the Cochrane Handbook [99]. The protocol was registered on PROSPERO (International Prospective Register of Systematic Reviews; Ref CRD42021244192). The components and phases of the systematic review are shown in the PRISMA checklist (see Table S1 of the Supplementary Materials) and the flowchart (see Figure 1), respectively. The methodology followed is discussed in the following subsections.

### 3.1. Search Strategy

Five databases were used to search studies: PubMed-Medline, Ebsco, The Cochrane Library, CINAHL, and Web of Science. Additionally, we used ProQuest and Google Scholar to search grey literature. To develop the search strategy (see Table S2 of the Supplementary Materials), a pilot search was conducted following the Peer Review of Electronic Search Strategies (PRESS) [100] and the guidelines proposed by the Effective Practice and Organisation of Care (EPOC) group [82]. In addition, we used terms associated with the PICOS elements (population, intervention, comparator, outcome, and study design). In order to follow up on the progress of LH and DT interventions, studies published in English up to June 2022 were searched. Identifying relevant supplemental literature was completed by reviewing the references from the acquired studies.

### 3.2. Selection of Studies

Controlled before–after (CBA) studies and randomized controlled trials (RCTs) were included. Additionally, case-control, pre-post, and cohort studies were included to generalize the effect of the interventions. We included studies carried out in healthcare units applying the intervention to inpatient and outpatient care, including primary to quaternary care within the public or private sector. Included interventions took place in one or more departments.

We selected studies addressing LH interventions (i.e., lean system, lean thinking, or Toyota production system) and LH-related tools and principles. DTs used by healthcare organizations to manage healthcare delivery included technologies related to Industry 4.0 and those explicitly mentioning the adoption of Healthcare 4.0. They encompass the IoT, artificial intelligence, big data, cloud computing, fog or mobile computing, virtual and augmented reality, robotics, remote sensing, 3D printing, automation, simulation, open-source software, and blockchain.

As identified in previous systematic reviews of LH interventions [94,101,102], we searched for studies reporting main outcomes related to patient flow, such as those described as utilization or access to services by the Cochrane EPOC Group [82]. However, to expand our findings, we also searched for studies reporting quality of care and patient outcomes [82]. Utilization of services outcomes included (i) length of stay (LOS) for outpatient, measured as the time a patient goes from admission to discharge; (ii) length of stay for inpatients (the time from occupying a bed until the time of discharge from the hospital); (iii) turnover time (TOT), the time between the departure of one patient and the arrival of the next one; and (iv) turnaround time (TAT), the time from completing one procedure or service to the beginning of a subsequent procedure or service. Outcomes related to access to services included (i) boarding time, measured as the interval elapsed between the admission decision and the assignment of a hospital bed; (ii) waiting time, the time spent waiting for a consultation completed by a health professional; (iii) number of individuals who left without being seen (LWBS); and (iv) the waiting time for an appointment. For patient outcomes, we searched for those related to health status, such as mortality rate. We searched for quality of care: (i) readmission rate, measured as the percentage of patients who experienced unplanned readmissions to a hospital after a previous hospital stay, and (ii) adherence to recommended guidelines or practices. Changes in patient satisfaction and staff satisfaction were searched as secondary outcomes, measured as an average satisfaction score. We included satisfaction data gathered using validated instruments such as the Patient Satisfaction Questionnaire (PSQ-III), the HCAHPS survey [103], and the Picker Patient Experience Questionnaire (PPE-15).

### 3.3. Data Extraction, Synthesis, and Risk of Bias

Two reviewers screened each study independently in regard to title, abstract, and keywords to identify its contribution and research context to consider it for an in-depth evaluation. The percentage of disagreement was around 10% and was resolved by consensus. The complete text of pertinent studies was then evaluated by two reviewers concerning the inclusion/exclusion criteria. A third reviewer evaluated studies for which a consensus was not reached (around 5% of the cases). One reviewer extracted data from articles, and then the second reviewer checked the data. The extracted data included the study’s location, setting, duration, aims, design, population, intervention, and control conditions, among other relevant study characteristics reported in similar studies [94,101]. The screening, evaluation, and extraction activities were performed manually, using reference manager software and a spreadsheet. Finally, all data were tabulated utilizing standardized forms. Due to the heterogeneity in the studies and the lack of RCTs, results could not be pooled to perform a meta-analysis. Instead, we conducted a descriptive synthesis of the results, following similar approaches [43,44,48], and summarized the findings of the main outcomes by utilizing the reported effect measures in each study (percentages, medians, and means).

The majority of the studies included were observational. Therefore, the risk of bias was assessed by employing Cochrane’s tool ROBINS-I (Risk Of Bias In Non-randomized Studies of Interventions) [104,105]. The judgment criteria comprised seven bias domains with five levels (low, moderate, serious, critical, and no information) [104]. To reach an overall judgment of the risk of bias, two reviewers independently evaluated each study following the ROBINS-I algorithm; consensus was obtained through a third reviewer’s assessment when a difference persisted.

## 4. Results

The search in the databases yielded 4052 titles. After removing duplicates, opinion papers, and abstracts, 1015 studies remained for the screening. Then, 813 studies were removed after applying the criteria for exclusion, leaving 202 possible studies submitted for eligibility. During the full-text review stage, 174 studies were eliminated based on the criteria in Table 1. In this review, 28 studies were considered. Figure 1 depicts the identification, screening, and inclusion process. In addition, Table S3 of the supplementary material provides an extended summary of findings. EDs are the most recurrent setting for interventions, appearing in 10 studies, followed by laboratories (5 studies) and operating rooms (5 studies). The United States is the country where more interventions took place (17 studies), which is consistent with previous studies reviewing only LH interventions [94,101] and a survey documenting LH and similar interventions in 70% of American hospitals [106].

**Figure 2:**
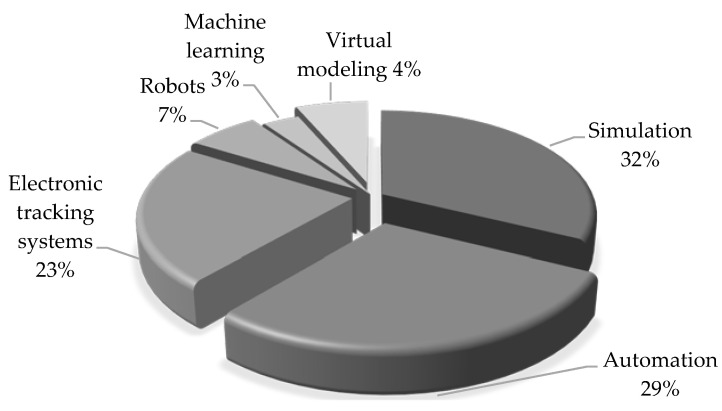
Main digital technologies used to support lean and Six Sigma interventions.

Simulation and automation were the leading supportive digital technologies used in the LH interventions. In 10 studies, simulation yielded improvements in 17 out of the 18 reported outcomes, with the one remaining outcome reporting no improvement [107]. Automation was the supportive technology in 9 studies, yielding improvements in 15 out of 17 outcomes. In seven studies, electronic tracking systems were utilized as supportive technology, which reported improvements in 10 outcomes and no significant change in one outcome [108]. Less frequently used technologies included robots to improve the turnaround time and turnover time [69,93], machine learning and simulation to improve LOS and LWBS [20], and virtual modeling combined with simulation to improve patients’ waiting times [109,110], as shown in Figure 2.

TAT was the most frequent metric, with 12 studies reporting a reduction in all 17 outcomes after interventions. Laboratories were the most recurrent setting in which TAT was measured (5 studies), all reporting a reduction in TAT; the largest reductions were 31.3 min in a clinical lab study [111] and 10 days in a histopathology lab study [112]. Table 2 shows all TAT outcomes and, when available, statistics from the studies.

Eleven studies reported 20 outcomes associated with patients’ length of stay (LOS), 16 of them reporting a decrease after the intervention (see Table 3). Conversely, three studies reported no change after the intervention [35,107,108], and one reported an increased LOS [107]. Within LOS outcomes for ambulatory patients, the most significant reduction was from 5.8 h to 4.1 h [119], whereas for inpatients, the most considerable reduction was from 22.9 days to 13.2 days [33].

Concerning waiting times, six studies reported improvements in all six outcomes (see Table 4). The largest reduction in waiting time was from 201.6 min to 103.1 min, occurring in ED [110]. In addition, one study reported an improvement in the percentage of patients seen within 30 min, increasing from 33% to 93% after the intervention [121].

Five studies reported a reduction in four out of six TOT outcomes; the largest (65 min) reduction occurred at a pediatric facility [69]. On the contrary, two non-significant increases in TOT after the intervention were reported [33,93]. TOT was measured in various settings, including pediatric facilities [69], ambulance services [125], pharmacies [33], and operating rooms [93,126]. Table 5 depicts the findings of TOT outcomes.

Four studies measured the percentage of LWBS. Each reported reductions, with the largest (30%) taking place in an emergency care setting [20]. Table 6 depicts the findings related to LWBS.

We found two studies measuring the 30-day readmission rate, one reporting a reduction [20] and another a non-significant change [108]. In addition, we found one study that measured the adherence to recommended practices, reporting a proportion increase in appropriate perioperative antibiotics therapy among surgical patients (from 25.5% to 44%; *p* < 0.01) [35]. Finally, we did not identify any outcome related to boarding time, mortality rate, and waiting time for an appointment. However, we did find additional outcomes related to resource use, including an improvement in the percentage of on-time starts in the OR [93], staff walking distance and nurse lead time [118], room utilization and overtime [93], hospitalization cycle time [120], and the decrease in the number of surgical instruments as well as improvements in the Mayo setup time [116]. Only two studies evaluated patient satisfaction, each reporting improvements after the intervention [119,127]. Staff satisfaction was measured in one study, reporting increases associated with the emergency room and lab [127].

**Figure 3:**
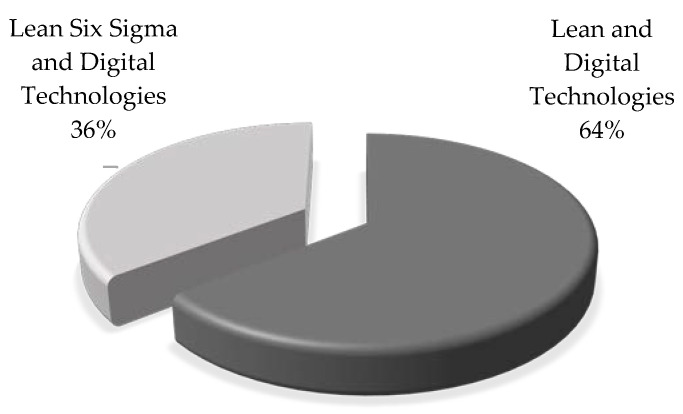
Main interventions supported by digital technologies.

Regarding the types of interventions, 18 studies utilized LH supported by one or more DTs. The remaining 10 studies reported a combination of LH and Six Sigma interventions, supported by at least one DT (Figure 3). Regarding the research scope, all interventions occurred in departments or processes but not in the whole organization. Regarding the risk of bias, Table S4 of the supplementary material depicts the assessments of the studies. Four interventions were assessed with low bias, 22 with moderate bias, and two with serious bias.

## 5. Discussion

### 5.1. Effects of LH and DT Interventions on Healthcare Services

According to our results, most LH interventions supported by DTs have a positive effect on outcomes oriented to patient flow (TAT, LOS, TOT, waiting time, and LWBS). Therefore, LH and DTs best serve to improve outcomes related to the utilization, coverage, or access to services, as well as resource use. Conversely, LH and DTs have less focus on patient outcomes (health, safety, and satisfaction), staff outcomes, and savings. Moreover, we did not find evidence on outcomes related to mortality rate, boarding time, and appointment waiting time. We anticipated that a reduction in TAT or TOT might decrease waiting times for patients and staff, which might contribute to a reduction in the percentage of LWBS and, ultimately, a reduction in LOS. Despite this inherent relationship among outcomes, we did not find studies focusing on a cause–effect analysis.

Although patient satisfaction is key in directing process improvement initiatives in healthcare systems [128,129,130], it is rarely measured and monitored in LH–DT interventions. This finding is consistent with previous studies highlighting that most LH interventions tracked the effects on throughputs but disregarded patient satisfaction outcomes [94,101,131]. However, the finding is also contradictory, as patient involvement grows in relevance [132], given the continuous feedback facilitated by the IoT (e.g., social networks) [22]. Another outcome scarcely measured is staff satisfaction; we only identified one study [127]. A growing body of literature outlines the staff’s fundamental role in healthcare [133,134,135], highlighting the need for analytical attention and technological solutions focused on minimizing the burden experienced by physicians and nurses [134]. The findings imply that most intervention studies still focus on efficiency improvement in a department rather than taking a holistic perspective to optimize the outcomes across the entire patient journey process.

Several countries have adopted strategies to increase healthcare efficiency, including LH. For example, in the United States, less Medicare spending per beneficiary has been linked with LH interventions [136]. Despite being at the core of lean interventions, outcomes related to savings and earnings were reported in only three studies (see Table S3 of the Supplementary Materials). The lack of cost-related outcomes is consistent with reports of a negative association between LH and financial costs [102] and the inability displayed by hospitals to translate LH benefits into savings [101]. Multidisciplinary intervention teams could address these problems. Moreover, the cost reduction analysis possibly related to Healthcare 4.0 implementation [137] in LH interventions might be complemented by different investment decisions in such technologies [138].

### 5.2. Digital Technologies Supporting Lean Healthcare Interventions

Overall, simulation and automation were the main supportive digital technologies reported in LH interventions. Simulation was used mainly in ED settings for measuring patient-flow outcomes. Simulation stands out as a powerful decision-support technique among the main trends of the Industry 4.0 era [139]. We identified two approaches to the use of simulation in LH projects: (i) practical interventions using simulation as a means to model different scenarios in a healthcare setting followed by the adoption of the best solution, and (ii) theoretical studies that combine LH and simulation to propose potential solutions, with no reported implementation [130,140,141,142,143,144,145,146,147,148,149,150,151,152].

On the other hand, automation was used as supportive technology in nine LH interventions. Lean automation incorporates digital automation technologies into the operationalization of lean practices [153]. Our analysis found that automation was mainly used to support LH in laboratories [68,111,112,113] and radiology settings [90,124]. That also implies that healthcare can benefit from automation where standardized processes and routine operations are followed. In this regard, to obtain maximum benefits from automation, it is reasonable to first optimize the process through LH or Six Sigma [154].

Particularly, most of the LH and Six Sigma interventions followed the DMAIC approach (define, measure, analyze, improve, and control), which provides a formal and logical sequence for understanding the process and identifying opportunities for improvement [155,156,157,158].

Electronic tracking systems were another recurrent technology supporting LH, aimed at obtaining and transmitting data to examine the flow of staff, patients, and material. Less frequently reported technologies were robots [69,93], machine learning [20], and virtual modeling [109,110]. Although LH interventions are reportedly used in healthcare systems in different contexts [65], we did not find evidence of LH interventions supported by technologies such as IoT, big data, virtual and augmented reality, fog/mobile computing, cloud computing, 3D printing, telemedicine, open-source software, or blockchain. Although technologies such as AI, big data, telehealth, and cloud computing have been implemented in healthcare [159,160], there is scarce evidence that these technologies support LH interventions. Thus, few DTs have been utilized to support LH, reflecting a low pervasiveness in different healthcare settings [49]; however, that seems to signal initial approaches to incorporating, in a staggered manner, extended and more complex types of DTs into LH interventions. Particularly, simulation is considered an intermediate step towards more sophisticated technology such as digital twins, identified as the next modeling, simulation, and optimization paradigm [161,162]. In such a new paradigm, the use of simulation will be extended [161] to support the design and redesign of patient-centered healthcare settings.

Previous studies have classified DTs [27,31,137] into two groups according to their main purposes. The first group, named Processing–Actuation, included technologies that process data producing information to control a system or mechanism. In our review, simulation and automation best represented this group, followed by robot systems, virtual modeling, and machine learning. The second group, called Sensing–Communication, included technologies for capturing and communicating data. In our review, electronic tracking systems best represented this group. Moreover, based on the effects of LH interventions supported by DTs, we identified three primary beneficiaries: patients (11 studies), resource management (12 studies), and healthcare professionals (3 studies). However, as previously suggested [30], we did not identify studies focused on high-level healthcare systems.

### 5.3. Settings and Challenges

In regards to our results, the largest number of interventions occurred in EDs (10 studies), consistent with findings in Po et al. [136]. This may be justified by the relevance of EDs within the hospital structure and their well-reported overcrowding problems [163,164]. EDs also offer a controlled environment for experimentation and, together with intensive care units, are likely to benefit the most from DTs [165]. Laboratories followed, with five reported interventions. Although system-wide healthcare management processes require more lean approaches and higher reliance on technology to achieve optimized operations and lower costs [166], all interventions in this review focused on particular processes or departments, which is aligned with the literature on lean in healthcare [46,167]. Small and focused improvements support the organization’s ability to sustain momentum, and early achievements are key to keeping people from becoming dispirited [93]. Hospital-wide improvements require not only broad and sustained commitment [167] but also capital expenditures and a more skilled labor force [49]. Future studies need to focus on how LH interventions supported by DTs could improve system-level outcomes rather than focusing on wards.

LH and DTs as a dual intervention entail significant technical and organizational challenges, including the reallocation of labor and equipment resources [124]. Such reorganization is needed to adapt the technology to social needs, including the beliefs and barriers of patients and caretakers [168], as well as cultural barriers, lack of awareness, and resource limitations [160]. Moreover, physicians’ resistance to change has been commonly reported when implementing lean interventions [169,170,171] due to the perception that lean interventions might target established medical practices [169]. However, in the present study, we did not identify any reported resistance to the dual intervention of LH and DTs. This could become a future research avenue due to the increasing implementation of these interventions.

Regarding technical challenges, IT services and infrastructure present various implementation difficulties [172], including difficulties with system configuration, system access, software updates, and poor user interfaces [173], all identified as sources of delayed care [53]. Further difficulties in implementing DTs included a negative impact on staff communication and the level of situational awareness in EDs in converting to automated tracking systems from manual ones [174]. The age and ownership of the hospital, type of hospital, number of inpatient beds, and number of employees have also been reported as contingency factors in adopting Healthcare 4.0 technologies [49]. In this regard, the literature evidence related to difficulties with DTs in healthcare remains mainly qualitative [53] and is scarce in the context of LH.

Based on our findings, LH and DTs have a complementary effect. This is consistent with previous studies in manufacturing settings [55,57]. We anticipate two different approaches to LH interventions supported by DTs. The first is a sequential approach in which LH is implemented to improve the process flow, followed by the adoption of supporting suitable DTs. Through such an approach, the full potential of technology integration can be realized by ensuring the elimination of needless legacy tasks [124]. In the second approach, LH and DTs are simultaneously implemented, requiring more resources and capabilities from the organization. Simulation and digital twins might contribute to supporting such an approach, anticipating different implementation scenarios.

### 5.4. Study Limitations

Due to the nature of our research, some limitations are present. In the first place, observational pre–post designs prevailed among the studies. Thus, the possible existence of confounding variables and the lack of randomization prevented us from determining a cause–effect relationship between the interventions and the outcomes. Furthermore, differences in data (settings, length of the studies, data collection, and processing approaches) dictate that care be taken not to generalize the results of our research. Finally, the risk of bias and heterogeneity of studies prohibited us from performing a meta-analysis.

## 6. Conclusions

Most interventions of LH supported by DTs reported a significant positive effect on one or more outcomes related to patient flow, namely, TAT, LOS, waiting time, TOT, and patient LWBS. However, there is scarce evidence of the effects of the interventions on other outcomes associated with patients (health, satisfaction, and safety), staff, quality of care, resource use, and savings. Most LH interventions used simulation or automation as the main supportive technology, and EDs and laboratories were recurrent settings. The interventions may be viewed as initial attempts toward incorporating a wide variety of settings and more complex DTs. Therefore, more studies focusing on patient outcomes, quality of care, resource use, and staff outcomes are required to shed light on adapting, implementing, and integrating DTs into interventions such as LH.

One-third of the analyzed interventions utilized the LH and Six Sigma approach in combination with DTs, reporting twofold benefits on healthcare. LH and Six Sigma benefit from the massive and faster data collection and analysis that Healthcare 4.0 DTs provide, leading to the timely identification of root causes of variation and waste generation. Conversely, DTs benefit from the structured approach of LH and Six Sigma to solve efficiency and variation problems and as a foundation for streamlining work and stabilizing processes before implementing more sophisticated or expensive technologies.

When efficiency and cost consideration play a significant role in the decision-making process, healthcare services are challenged to apply compliance strategies without compromising the quality of healthcare. In this regard, the benefits of implementing LH and DTs should outweigh the implementation efforts. Different investment decisions might complement the cost reduction analysis of using DTs to support LH on such technologies. Therefore, more effective planning and preparation can occur once organizations recognize the dual LH–DT challenges.

## 7. Future Research

Based on the results and the discussion, we identified some gaps related to incorporating DTs into LH initiatives. First, more evidence is needed to describe problems and benefits of DTs in healthcare in the context of LH interventions. Second, more studies focusing on patient and staff outcomes (health, safety, and satisfaction) are required. Third, future studies should expand the research by analyzing the effect that settings have on the use of technology. Fourth, due to the LH focus on waste reduction, further research on LH supported by DTs and their combined effect on sustainability is also required. In addition, some directions for future research on LH and DTs include:

### 7.1. Applications of Sensing–Communication Technologies in LH Interventions

A bundle of five DTs was grouped under the label “Sensing–Communication” in previous work [137]. They are big data, IoT, biomedical/digital sensors, cloud computing, and remote control or monitoring. As reported earlier, electronic tracking systems and sensors, which belong to the remote-control category, are the sensing–communication DTs used in LH interventions. They are used to track and monitor patients in ED and surgical theaters. We envision two promising applications of sensing–communication DTs in LH improvement projects. The first one applies RFID (radio-frequency identification) for surgical instruments to control the quality of surgical tray assembly and instrument traceability. Trays with missing instruments are a recurrent problem in surgical theaters [175]; an LH project to adopt electronic Poka–Yokes (mistake proofing) at the final stage of tray assembly would help to address the problem. The second one uses biomedical sensors connected to the IoT to track inward patients’ vital signals, activate rapid-response teams, and reduce the time to assist patients in critical condition. A previous study [176] showed that technology may play a major role in improving the performance of those teams, which could be analyzed through an LH project.

### 7.2. Electronic Kanbans to Improve the Management of Patient Transportation

Patient transportation inside hospitals poses a serious constraint on the efficient operation of several processes, such as patient admission and discharge [177]. Previous studies have proposed the use of tools such as spaghetti charts, value-stream mapping, activity worksheets [178], and the single-minute exchange of die (SMED) [179]. In this research opportunity, we propose using electronic Kanbans to prioritize patients to be transported according to a set of criteria established by the hospital. The electronic transportation Kanban could be incorporated into the hospital system such that online updates become possible, being accessible by the transportation team throughout the hospital. Incorporating RFID tags on the transportation equipment (e.g., stretchers and wheelchairs) is another use of DTs likely to improve the performance of the Kanban-operated transportation management system.

### 7.3. Virtual Reality Enabling Lean Layout Studies

The study of industrial layouts through lean methods has been previously reported in the literature, e.g., Nagi and Altarazi [180] and Fogliatto et al. [181]. However, the layouts are based on manually implemented lean tools, such as value-stream mapping and spaghetti charts. Healthcare layout design should consider alternatives’ impacts on patients and staff flows and their related metrics. The use of virtual reality as a supporting tool in LH layout design projects is likely to produce more efficient layouts. An initial step in the direction of a virtual reality-based framework for designing healthcare layouts could be the adaptation of the proposition in Zhi-hua and Yi-fang [182], which addresses the manufacturing layout problem.

## Figures and Tables

**Table 1:** Systematic review framework.

Process	Criteria	Description
Search strategy	Data sources	PubMed-Medline, Ebsco, The Cochrane Library, CINAHL, Web of Science, ProQuest, and Google Scholar
	Studies	Studies published in English up to June 2022
Selection of studies	Participants	Healthcare units (inpatient and outpatient) providing direct service to patients Primary to quaternary care
	Intervention	Lean methodologies and similar interventions Industry 4.0 digital technologies
	Comparator	Effect measures (mean, median, or percentages) of pre- vs. post-intervention or control group vs. intervention group
	Outcomes	Patient outcomes, quality of care, utilization and access to service, resource use, patient and staff satisfaction
	Study design	Randomized control trials, controlled before–after, pre–post, case-control, cohort
	Exclusion criteria	Surveys, reviews, opinion papers, technical notes, interviews, and editorial letters Studies published in languages other than English Studies that did not include a patient-oriented or direct healthcare service (e.g., suppliers’ efficiency, administrative staff efficiency, medical device efficiency, the efficiency of a medical device manufacturing company) Studies without abstract and data
Data extraction and synthesis	Review process Extracted data	Two reviewers screened, assessed, and extracted data. A third reviewer assessed when consensus was necessary. Study location, settings, duration, aims, design, participants, intervention, comparator, outcomes, findings, and control conditions
Risk of bias	Tool	Cochrane Risk of Bias in Non-randomized Studies of Interventions (ROBINS-I)

**Table 2:** TAT outcomes of LH interventions supported by DTs.

(Authors, Year) Country	Settings; Study Design; *n*; Time Frame	Intervention	Main Outcomes	Summary of Findings
(Wongkrajang, 2020) [68] Thailand	Laboratory; case study, pre–post; *n* = 30,180; 3 mo	Lean and automation	90th-percentile TAT	Reduced from 60 min to 50 min (*p* = 0.01)
(Ankrum, 2019) [69] USA	pediatric facility; case study, pre–post; *n* = 47 room turnovers; 60 days	Lean, robotics, and electronic medical records	Median time between room breakdown to cleaning start time	Reduced from 10 min to 3 min (*p* = 0.004)
(Recht, 2019) [90] USA	MRI; case study, pre–post; *n*1 = 5461 and *n*2 = 9221; 6 mo	Lean and automation of MRI (software tool)	Mean TAT (patients ready for scanning)	Reduced from 328 min to 132 min (*p* < 0.001)
			Mean TAT (all patients assessed)	Reduced from 537 min to 272 min (*p* < 0.001)
(Shilpasree, 2019) [111] India	Clinical laboratory; pre–post; *n* = 3344; 2 mo	Lean, Six Sigma, automation and computerization	TAT	Reduced from 110 min to 78.7 min (*p* < 0.001)
(Jensen, 2019) [113] USA	Laboratory; pre–post; *n* = 21,639; 20 mo; 4 mo follow up	Lean and automation (automated chemistry line and barcoding)	Specimen TAT	TnT: reduced from 56.64 min to 53.68 min (*p* < 0.001)
				K+: reduced from 40.88 min to 39.82 min (*p* < 0.001)
				CMP-Alb: reduced from 43.44 min to 40.51 min (*p* < 0.001)
(Brunsman, 2018) [33] USA	Inpatient pharmacy; cohort study; *n* = 102; 15 mo	Lean and automation of dispensing cabinet	Median overall TAT from CMS-approved antibiotic order entry to medication administration	Reduced from 120 min to 80 min (*p* = 0.014)
(Bhat, 2016) [114] India	Medical record department; case study; *n* = 100; 2 mo	Lean, Six Sigma and simulation	TAT of medical record preparation	Reduced from 19 min to 8 min
(Thureson, 2015) [112] Sweden	Histopathology lab; pre–post; *n* = 46,675; 27 mo	Lean and automatic embedding console	Median TAT for patients with breast cancer	Reduced from 25 days to 15.5 days (*p* < 0.001)
(Sanders, 2015) [115] USA	ED, hematology lab, and chemistry lab; pre–post	Lean, Six Sigma, ED tracking boards, electronic orders, and EHR	Median TAT for ED specimens of complete blood count analysis	Reduced from 15 min to 11 min
(Wannemuehler, 2015) [116] USA	OR; pre–post; *n* = 644; 10 mo	Lean, Six Sigma and electronic tracking system	Median assembly times (instrument set)	Reduced from 8.4 min to 4.7 min (*p* < 0.001)
			Mean Mayo setup times	Reduced from 97.6 s to 76.1 s (*p* < 0.001)
(White, 2014) [117] USA	ED; prospective controlled; pre–post; *n* = 59,687; 17 mo	Lean, Six Sigma, QT, TOC, and electronic patient tracking system	Median exam room time	The intervention group reduced by 34 min from 90 to 56 min (*p* < 0.001). The control group increased from 28 min to 36 min (*p* < 0.001).
(Nelson-Peterson, 2007) [118] USA	General hospital; time-series; pre–post; *n* = 8; 5 mo	Lean and simulation	Registered nurse lead time	Reduced from 240 min to 126 min
			Setup time (minutes for one cycle of care)	Reduced from 20 min to 3 min

Note. CMP-Alb indicates complete metabolic panel albumin; CMS, centers for Medicare and Medicaid services; ED, emergency department; EHR, electronic health record; h, hours; K+, potassium; min, minutes; mo, months; MRI, magnetic resonance imaging; OR, operating room; QT, queuing theory; RAD, rapid assessment and disposition process; TAT, turnaround time; TOC, theory of constraints; TPS, Toyota production system; TnT, troponin.

**Table 3:** LOS outcomes of LH interventions supported by DTs.

(Authors, Year) Country	Settings; Study Design; *n*; Time Frame	Intervention	Main Outcomes	Summary of Findings
(Tsai et al., 2021) [120] Taiwan	Operating room; case study, pre–post; *n* = 2964; 24 mo	Lean, Six Sigma, electronic tracking system (electronic tags, registration	Mean LOS	Orthopedic surgery Reduced from 3.31 days to 1.57 days
			Mean LOS	Colon and rectal surgery Reduced from 2.49 days to 1.16 days
		APP, QR-codes, perioperative flow system, and HIS	Mean LOS	Urology surgery Reduced from 3.31 days to 1.57 days
			Mean LOS	Otorhinolaryngology surgery Reduced from 2.49 days to 1.16 days
(Brunsman, 2018) [33] USA	Inpatient pharmacy; cohort study; *n* = 102; 15 mo	Lean and automation of dispensing cabinet	Median LOS	Reduced from 22.9 days to 13.2 days (*p* = 0.049)
(Rutman, 2015) [121] USA	ED; pre–post; *n* = 98; 7 mo	Lean, simulation, and EMR	Mean LOS in ED	Reduced by 30 min
(Beck, 2015) [108] USA	Inpatient pediatric service; pre–post; *n* = 3509; 12 mo	Lean, Six Sigma and tele-tracking systems	Mean LOS	Non-significant change, from 3.1 days to 3.0 days (*p* = 0.864)
(Lee, 2015) [20] USA	Emergency care center; *n* = 18,726; 9 mo	Process mapping, machine learning, simulation, and optimization	Overall LOS	Reduced from 10.59 h to 7.14 h
(Lo, 2015) [107] USA	Pediatric ED; pre–post; 7 mo	Lean, real-time voice recognition system, simulation, electronic charting, and EHR	Ambulatory patients’ LOS	Increased from 161 min to 168 min
			Inpatients’ LOS	No change (270 min)
(Tejedor-Panchon, 2014) [122] Spain	ED; Quasi-experimental pre–post study; *n* = 256,628; 36 mo	Lean, simulation, and digital technology in X-ray	Mean LOS in ED (time spent in the examination area)	NUC reduced from 80.4 min to 61.6 min (*p* < 0.001); TC reduced from 137.8 min to 123.8 min (*p* < 0.05); MSC reduced from 219.7 min to 209.3 min (*p* = 0.108)
(White, 2014) [117] USA	ED; prospective controlled, pre–post study; *n* = 59,687; 17 mo	Lean, Six Sigma, QT, and TOC; electronic patient tracking system	Median LOS for ambulatory patients	Intervention group reduced from 158 min to 143 min (*p* < 0.001) Non-significant change in the control group, from 265 min to 267 min (*p* = 0.69)
(Furterer, 2014h [119] USA	ED; case study; 7 mo	Lean, Six Sigma, automation, electronic ED bed board, EMR	Mean LOS (all patients)	Reduced from 6.9 h to 4.7 h (*p* < 0.001)
			Mean LOS for inpatients	Reduced from 8.7 h to 6.1 h
			Mean LOS for ambulatory patients	Reduced from 5.8 h to 4.1 h
(Burkitt, 2009) [35] USA	Department of surgery; cohort study; *n* = 1779; 48 mo	TPS, automatic control of antibiotics after surgery, and computerized medical record	Median LOS	Non-significant change (*p* = 0.90)
(Eller, 2009) [123] USA	ED; pre–post; 25 mo	Lean, patient track, and electronic documentation system	Mean LOS for no RAD patients	Reduced 45 min
			Mean LOS for RAD patients	Reduced 208 min

Note. ED indicates emergency department; EMR, electronic medical records; h, hours; HIS, healthcare information system; LOS, length of stay; MSC, medical–surgical cases; min, minutes; mo, months; NUC, non-urgent circuit; QT, queuing theory; RAD, rapid assessment and disposition process; TC, trauma cases; TOC, theory of constraints; TPS, Toyota production system.

**Table 4:** Waiting time outcomes of LH interventions supported by DTs.

(Authors, Year) Country	Settings; Study Design; *n*; Time Frame	Intervention	Main Outcomes	Summary of Findings
(Ortiz-Barrios, 2020) [110] Colombia	ED; case study; *n* = 16,741; 15 mo	Lean, simulation and virtual modeling	Mean waiting time	Reduced from 201.6 min to 103.1 min
(Baril, 2016) [109] Canada	Hematology–oncology clinic; case study; 10 mo, 2 mo of follow up	Lean, simulation, and business game virtual environment	Mean patient waiting time before treatment	Reduced from 61 min to 16 min
(Rutman, 2015) [121] USA	ED; pre–post; *n* = 98; 7 mo	Lean, simulation, and electronic medical records	Median time to see a provider	Reduced from 43 min to 7 min
			Patients seen within 30 min	Increased from 33% to 93%
(Rico, 2015) [124] USA	ED; pre–post; *n* = 50; 1 mo	Lean and automated infusion system	Mean waiting time for FDG Infusion	Reduced from 11.3 min to 6.4 min (*p* < 0.01)
(Tejedor-Panchon) [122] Spain	ED; quasi-experimental pre–post study; *n* = 256,628; 36 mo	Lean, simulation, and digital technology in X-ray	Mean waiting time to see a physician	Reduced from 58.0 min to 49.1 min (*p* < 0.001)
(Furterer, 2014) [119] USA	ED; case study; 7 mo	Lean, Six Sigma, automation, electronic ED bed board, and EMR	Time from door to doctor	Reduced from 100 min to 27 min

Note. ED indicates emergency department; FDG, fluorodeoxyglucose; h, hours; min, minutes; mo, months.

**Table 5:** TOT outcomes of LH interventions supported by DTs.

(Authors, Year) Country	Settings; Study Design; *n*; Time Frame	Intervention	Main Outcomes	Summary of Findings
(Amati et al., 2022) [126] Switzerland	Operating room; case study, pre–post; 9 mo	Lean and simulation	Mean surgery changeover time (skin to skin)	Gynecological surgery Reduced from 58 min to 41 min
			Mean surgery changeover time (skin to skin)	General surgery Reduced from 63 min to 48 min
(Ankrum, 2019) [69] USA	Pediatric facility; case study, pre–post; *n* = 47 room turnovers; 60 days	Lean, robotics, and electronic medical records	Median room turnover time	Reduced from 130 min to 65 min (*p* < 0.001)
(Garza-Reyes, 2019) [125] Mexico	Ambulance service; case study; *n* = 850 ambulances; 1 mo	Lean and simulation, internet-based technologies, and GPS tracking devices	Average ambulance cycle time	Reduced from 124.9 min to 75.8 min
(Brunsman, 2018) [33] USA	Inpatient pharmacy, cohort study; *n* = 102; 15 mo	Lean and automation of dispensing cabinet	Median time from order to medication verification	Increased from 5.5 min to 10.5 min (*p* = 0.11)
(Bender, 2015) [93] USA	Operating room; pre–post; *n* = 25,903; 36 mo	Lean, Six Sigma, and robots	Mean turnover time	Non-significant change from 43 min to 44 min

Note. GPS indicates global positioning system; min, minutes; mo, months.

**Table 6:** Outcomes of LWBS in LH interventions supported by DTs.

(Authors, Year) Country	Settings; Study Design; *n*; Time Frame	Intervention	Main Outcomes	Summary of Findings
(Lee, 2015) [20] USA	Emergency care center; *n* = 18,726; 9 mo	Process mapping, machine learning, simulation, and optimization	Percentage of patients LWBS	Reduced by 30%
(Tejedor-Panchon, 2014) [122] Spain	ED; quasi-experimental pre–post study; *n* = 256,628; 36 mo	Lean, simulation, and digital technology in X-ray	Percentage of patients LWBS	Reduced from 2.8% to 2.0% (*p* < 0.001)
(Furterer, 2014) [119] USA	ED; case study; 7 mo	Lean, Six Sigma, electronic ED bed board, electronic medical record, and automation	Percentage of patients LWBS	Reduced from 6.5% to 0.34 %
(Eller, 2009) [123] USA	ED; pre–post; 25 mo	Lean, patient track, and electronic documentation system	Percentage of patients LWBS	Reduced 28%

Note. ED indicates emergency department; LWBS, left without being seen; mo, months.

## Data Availability

Not applicable.

## Supplementary Materials

The following supporting information can be downloaded at https://www.mdpi.com/article/10.3390/ijerph19159018/s1, Table S1: PRISMA Checklist. Table S2: Search strategy; Table S3: Extended summary of findings; Table S4: Risk of bias.

## References

[1] Yu T., Demirli K., Bhuiyan N. (2021). Lean transformation framework for treatment-oriented outpatient departments. Int. J. Prod. Res..

[2] De Barros L.B., Caldas L.P., Bohomol E., Sarantopoulos A., Minatogawa V., Gasparino R.C. (2022). Evaluation of Waste Related to the Admission Process of Low-Complexity Patients in Emergency Services, in Light of the Lean Healthcare Philosophy. Int. J. Environ. Res. Public Health.

[3] Beck M., Okerblom D., Kumar A., Bandyopadhyay S., Scalzi L. (2016). Lean intervention improves patient discharge times, improves emergency department throughput and reduces congestion. Hosp. Pract..

[4] Sánchez M., Suárez M., Asenjo M., Bragulat E. (2018). Improvement of emergency department patient flow using lean thinking. Int. J. Qual. Health Care J. Int. Soc. Qual. Health Care.

[5] Shortell S., Bennett C., Gayle B., Byck G. (1998). Assessing the Impact of Continuous Quality Improvement on Clinical Practice: What It Will Take to Accelerate Progress. Milbank Q..

[6] Sirvent J.M., Gil M., Alvarez T., Martin S., Vila N., Colomer M., March E., Loma-Osorio P., Metje T. (2016). Lean techniques to improve flow of critically ill patients in a health region with its epicenter in the intensive care unit of a reference hospital. Med. Intensiva (Engl. Ed.).

[7] Hseng-Long Y., Chin-Sen L., Chao-Ton S., Pa-Chun W. (2011). Applying lean six sigma to improve healthcare: An empirical study. Afr. J. Bus. Manag..

[8] Gayed B., Black S., Daggy J., Munshi I.A. (2013). Redesigning a Joint Replacement Program using Lean Six Sigma in a Veterans Affairs Hospital. JAMA Surg..

[9] Cromwell S., Chiasson D.A., Cassidy D., Somers G.R. (2018). Improving Autopsy Report Turnaround Times by Implementing Lean Management Principles. Pediatr. Dev. Pathol..

[10] Van Vliet E.J., Sermeus W., Van Gaalen C.M., Sol J.C.A., Vissers J.M.H. (2010). Efficacy and efficiency of a lean cataract pathway: A comparative study. Qual. Saf. Health Care.

[11] Hydes T., Hansi N., Trebble T.M. (2012). Lean thinking transformation of the unsedated upper gastrointestinal endoscopy pathway improves efficiency and is associated with high levels of patient satisfaction. BMJ Qual. Saf..

[12] Blackmore C., Kaplan G. (2017). Lean and the perfect patient experience. BMJ Qual. Saf..

[13] McDermott A., Kidd P., Gately M., Casey R., Burke H., O’Donnell P., Kirrane F., Dinneen S.F., O’Brien T. (2013). Restructuring of the Diabetes Day Centre: A pilot lean project in a tertiary referral centre in the West of Ireland. BMJ Qual. Saf..

[14] Halim U.A., Khan M.A., Ali A.M. (2018). Strategies to Improve Start Time in the Operating Theatre: A Systematic Review. J. Med. Syst..

[15] Pennathur P.R., Cao D., Bisantz A.M., Lin L., Fairbanks R.J., Wears R.L., Perry S.J., Guarrera T.K., Brown J.L., Sui Z. (2011). Emergency department patient-tracking system evaluation. Int. J. Ind. Ergon..

[16] Lin S., Gavney D., Ishman S.L., Cady-Reh J. (2013). Use of lean sigma principles in a tertiary care otolaryngology clinic to improve efficiency. Laryngoscope.

[17] Bendavid Y., Boeck H., Philippe R. (2012). RFID-enabled traceability system for consignment and high value products: A case study in the healthcare sector. Proc. J. Med. Syst..

[18] Tortorella G.L., Saurin T.A., Fogliatto F.S., Rosa V.M., Tonetto L.M., Magrabi F. (2021). Impacts of Healthcare 4.0 digital technologies on the resilience of hospitals. Technol. Forecast. Soc. Chang..

[19] Holtz B.E. (2021). Patients Perceptions of Telemedicine Visits before and after the Coronavirus Disease 2019 Pandemic. Telemed. E-Health.

[20] Lee E., Atallah H., Wright M., Post E., Thomas C., Wu D., Haley L. (2015). Transforming hospital emergency department workflow and patient care. Interfaces.

[21] Ibrahim M., Wedyan M., Alturki R., Khan M.A., Al-Jumaily A. (2021). Augmentation in Healthcare: Augmented Biosignal Using Deep Learning and Tensor Representation. J. Healthc. Eng..

[22] Arcidiacono G., Pieroni A. (2018). The revolution Lean Six Sigma 4.0. Int. J. Adv. Sci. Eng. Inf. Technol..

[23] De Mast J., Kemper B., Does R.J.M.M., Mandjes M., Van Der Bijl Y. (2011). Process improvement in healthcare: Overall resource efficiency. Qual. Reliab. Eng. Int..

[24] Marshall D., Burgos-Liz L., Ijzerman M., Crown W., Padula W., Wong P., Pasupathy K., Higashi M., Osgood N. (2015). Selecting a dynamic simulation modeling method for health care delivery research—Part 2: Report of the ISPOR dynamic simulation modeling emerging good practices task force. Value Health.

[25] Du X., Chen B., Ma M., Zhang Y. (2021). Research on the Application of Blockchain in Smart Healthcare: Constructing a Hierarchical Framework. J. Healthc. Eng..

[26] Sharma L., Chandrasekaran A., Boyer K.K., McDermott C.M. (2016). The impact of Health Information Technology bundles on Hospital performance: An econometric study. J. Oper. Manag..

[27] Aceto G., Persico V., Pescapé A. (2018). The role of Information and Communication Technologies in healthcare: Taxonomies, perspectives, and challenges. J. Netw. Comput. Appl..

[28] Gastaldi L., Corso M. (2012). Smart healthcare digitalization: Using ICT to effectively balance exploration and exploitation within hospitals. Int. J. Eng. Bus. Manag..

[29] Balouei Jamkhaneh H., Luz Tortorella G., Parkouhi S.V., Shahin R. (2022). A comprehensive framework for classification and selection of H4.0 digital technologies affecting healthcare processes in the grey environment. TQM J..

[30] Al-Jaroodi J., Mohamed N., AbuKhousa E. (2020). Health 4.0: On the Way to Realizing the Healthcare of the Future. IEEE Access.

[31] Tortorella G.L., Fogliatto F.S., Espôsto K.F., Mac Cawley A., Vassolo R., Tlapa D., Narayanamurthy G. (2022). Measuring the effect of Healthcare 4.0 implementation on hospitals’ performance. Prod. Plan. Control.

[32] Almutairi A.M., Salonitis K., Al-Ashaab A. (2019). Assessing the leanness of a supply chain using multi-grade fuzzy logic: A health-care case study. Int. J. Lean Six Sigma.

[33] Brunsman A. (2018). Using lean methodology to optimize time to antibiotic administration in patients with sepsis. Am. J. Health Pharm..

[34] Moo-Young J.A., Sylvester F.A., Dancel R.D., Galin S., Troxler H., Bradford K.K. (2019). Impact of a Quality Improvement Initiative to Optimize the Discharge Process of Pediatric Gastroenterology Patients at an Academic Children’s Hospital. Pediatr. Qual. Saf..

[35] Burkitt K., Mor M.K., Jain R., Kruszewski M., Mccray E., Moreland M., Muder R., Obrosky D.S., Mary S., Wilson M. (2009). Toyota production system quality improvement initiative improves perioperative antibiotic therapy. Am. J. Manag. Care.

[36] Hussey P., De Vries H., Romley J., Wang M., Chen S., Shekelle P., McGlynn E. (2009). A systematic review of health care efficiency measures: Health care efficiency. Health Serv. Res..

[37] Mazzocato P., Savage C., Brommels M., Aronsson H., Thor J. (2010). Lean thinking in healthcare: A realist review of the literature. Qual. Saf. Health Care.

[38] Crema M., Verbano C. (2017). Lean Management to support Choosing Wisely in healthcare: The first evidence from a systematic literature review. Int. J. Qual. Health Care.

[39] Tasdemir C., Gazo R. (2018). A systematic literature review for better understanding of lean driven sustainability. Sustainability.

[40] Terra J.D.R., Berssaneti F.T. (2018). Application of lean healthcare in hospital services: A review of the literature (2007 to 2017). Production.

[41] Dellifraine J., Langabeer J., Nembhard I. (2010). Assessing the evidence of six sigma and lean in the health care industry. Qual. Manag. Health Care.

[42] Holden R.J. (2011). Lean thinking in emergency departments: A critical review. Ann. Emerg. Med..

[43] Nicolay C., Purkayastha S., Greenhalgh A., Benn J., Chaturvedi S., Phillips N., Darzi A. (2012). Systematic review of the application of quality improvement methodologies from the manufacturing industry to surgical healthcare. Br. J. Surg..

[44] Mason S., Nicolay C., Darzi A. (2015). The use of Lean and Six Sigma methodologies in surgery: A systematic review. Surgeon.

[45] Andersen H., Røvik K.A., Ingebrigtsen T. (2014). Lean thinking in hospitals: Is there a cure for the absence of evidence? A systematic review of reviews. BMJ Open.

[46] D’Andreamatteo A., Ianni L., Lega F., Sargiacomo M. (2015). Lean in healthcare: A comprehensive review. Health Policy.

[47] Costa L., Godinho Filho M. (2016). Lean healthcare: Review, classification and analysis of literature. Prod. Plan. Control.

[48] Amaratunga T., Dobranowski J. (2016). Systematic Review of the Application of Lean and Six Sigma Quality Improvement Methodologies in Radiology. J. Am. Coll. Radiol..

[49] Tortorella G.L., Fogliatto F.S., Espôsto K.F., Vergara A.M.C., Vassolo R., Mendoza D.T., Narayanamurthy G. (2020). Effects of contingencies on healthcare 4.0 technologies adoption and barriers in emerging economies. Technol. Forecast. Soc. Chang..

[50] Rubbio I., Bruccoleri M., Pietrosi A., Ragonese B. (2020). Digital health technology enhances resilient behaviour: Evidence from the ward. Int. J. Oper. Prod. Manag..

[51] Aceto G., Persico V., Pescapé A. (2020). Industry 4.0 and Health: Internet of Things, Big Data, and Cloud Computing for Healthcare 4.0. J. Ind. Inf. Integr..

[52] Kumari A., Tanwar S., Tyagi S., Kumar N. (2018). Fog computing for Healthcare 4.0 environment: Opportunities and challenges. Comput. Electr. Eng..

[53] Kim M.O., Coiera E., Magrabi F. (2017). Problems with health information technology and their effects on care delivery and patient outcomes: A systematic review. J. Am. Med. Inform. Assoc..

[54] Crema M., Verbano C. (2016). Identification and development of Lean and Safety projects. Saf. Sci..

[55] Buer S.-V., Semini M., Strandhagen J.O., Sgarbossa F. (2021). The complementary effect of lean manufacturing and digitalisation on operational performance. Int. J. Prod. Res..

[56] Gupta S., Modgil S., Gunasekaran A. (2020). Big data in lean six sigma: A review and further research directions. Int. J. Prod. Res..

[57] Ciano M.P., Dallasega P., Orzes G., Rossi T. (2021). One-to-one relationships between Industry 4.0 technologies and Lean Production techniques: A multiple case study. Int. J. Prod. Res..

[58] Pinho C., Mendes L. (2017). IT in lean-based manufacturing industries: Systematic literature review and research issues. Int. J. Prod. Res..

[59] Lee J. Smart Health: Concepts and Status of Ubiquitous Health with Smartphone. Proceedings of the ICTC 2011.

[60] Scarpato N., Pieroni A., Di Nunzio L., Fallucchi F. (2017). E-health-IoT universe: A review. Int. J. Adv. Sci. Eng. Inf. Technol..

[61] Sudana D., Emanuel A.W.R. How Big Data in Health 4.0 Helps Prevent the Spread of Tuberculosis. Proceedings of the 2019 2nd International Conference on Bioinformatics, Biotechnology and Biomedical Engineering (BioMIC)-Bioinformatics and Biomedical Engineering.

[62] Chute C., French T. (2019). Introducing care 4.0: An integrated care paradigm built on industry 4.0 capabilities. Int. J. Environ. Res. Public Health.

[63] Hathaliya J.J., Tanwar S., Tyagi S., Kumar N. (2019). Securing electronics healthcare records in Healthcare 4.0: A biometric-based approach. Comput. Electr. Eng..

[64] Tanwar S., Parekh K., Evans R. (2020). Blockchain-based electronic healthcare record system for healthcare 4.0 applications. J. Inf. Secur. Appl..

[65] Tortorella G.L., Fogliatto F.S., Mac Cawley Vergara A., Vassolo R., Sawhney R. (2020). Healthcare 4.0: Trends, challenges and research directions. Prod. Plan. Control.

[66] Raja R., Mukherjee I., Sarkar B.K., Ali S. (2020). A Systematic Review of Healthcare Big Data. Sci. Program..

[67] Salleh S., Thokala P., Brennan A., Hughes R., Booth A. (2017). Simulation Modelling in Healthcare: An Umbrella Review of Systematic Literature Reviews. Pharmacoeconomics.

[68] Wongkrajang P., Reesukumal K., Pratumvinit B. (2020). Increased effectiveness of urinalysis testing via the integration of automated instrumentation, the lean management approach, and autoverification. J. Clin. Lab. Anal..

[69] Ankrum A.L., Neogi S., Morckel M.A., Wilhite A.W., Li Z., Schaffzin J.K. (2019). Reduced isolation room turnover time using Lean methodology. Infect. Control Hosp. Epidemiol..

[70] Simons F.E., Aij K.H., Widdershoven G.A.M., Visse M. (2014). Patient safety in the operating theatre: How A3 thinking can help reduce door movement. Int. J. Qual. Health Care.

[71] Javaid M., Haleem A. (2019). Industry 4.0 applications in medical field: A brief review. Curr. Med. Res. Pract..

[72] Montesarchio V., Grimaldi A.M., Fox B.A., Rea A., Marincola F.M., Ascierto P.A. (2012). Lean oncology: A new model for oncologists. J. Transl. Med..

[73] Yong P.L., Saunders R.S., Olsen L., Yong P., Saunders R., Olsen L., Institute of Medicine (U.S.) (2010). Roundtable on Value & Science-Driven Health Care. The Healthcare Imperative: Lowering Costs and Improving Outcomes.

[74] Damle A., Andrew N., Kaur S., Orquiola A., Alavi K., Steele S.R., Maykel J. (2016). Elimination of waste: Creation of a successful Lean colonoscopy program at an academic medical center. Surg. Endosc..

[75] White B.A., Yun B.J., Lev M.H., Raja A.S. (2017). Applying Systems Engineering Reduces Radiology Transport Cycle Times in the Emergency Department. West. J. Emerg. Med..

[76] Weaver A., Greeno C.G., Goughler D.H., Kathleen Yarzebinski M., Tina Zimmerman B., Carol Anderson L. (2013). The impact of system level factors on treatment timeliness: Utilizing the toyota production system to implement direct intake scheduling in a semi-rural community mental health clinic. J. Behav. Health Serv. Res..

[77] Umut B., Alipour P., Sarvari P.A. (2016). Applying lean tools in the clinical laboratory to reduce turnaround time for blood test results. Int. J. Adv. Sci. Eng. Technol..

[78] Chan H., Lo S., Lee L., Lo W., Yu W., Wu Y., Ho S., Yeung R., Chan J. (2014). Lean techniques for the improvement of patients’ flow in emergency department. World J. Emerg. Med..

[79] Westwood N., James-Moore M., Cooke M., Wiseman N., Westwood N., James-Moore M., Cooke M. Going Lean in the NHS. https://www.england.nhs.uk/improvement-hub/wp-content/uploads/sites/44/2017/11/Going-Lean-in-the-NHS.pdf.

[80] Cohen R.I. (2018). Lean Methodology in Health Care. Chest.

[81] Bercaw R. (2011). Taking Improvement from the Assembly Line to Healthcare: The Application of Lean within the Healthcare Industry.

[82] Cochrane Effective Practice and Organisation of Care (EPOC) What Outcomes Should Be Reported in Cochrane Effective Practice and Organisation of Care (EPOC) Reviews?. http://epoc.cochrane.org/resources/epoc-resources-review-authors.

[83] Toledo A., Carroll T., Arnold E., Tulu Z., Caffey T., Kearns L., Gerber D. (2013). Reducing liver transplant length of stay: A lean six sigma approach. Prog. Transplant..

[84] Trzeciak S., Mercincavage M., Angelini C., Cogliano W., Damuth E., Roberts B.W., Zanotti S., Mazzarelli A.J. (2018). Lean Six Sigma to Reduce Intensive Care Unit Length of Stay and Costs in Prolonged Mechanical Ventilation. J. Healthc. Qual..

[85] Hitti E.A., El-Eid G.R., Tamim H., Saleh R., Saliba M., Naffaa L. (2017). Improving Emergency Department radiology transportation time: A successful implementation of lean methodology. BMC Health Serv. Res..

[86] Murrell K.L., Offerman S.R., Kauffman M.B. (2011). Applying Lean: Implementation of a Rapid Triage and Treatment System. West. J. Emerg. Med..

[87] Artenstein A.W., Rathlev N.K., Neal D., Townsend V., Vemula M., Goldlust S., Schmidt J., Visintainer P., Albert M., Alli G. (2017). Decreasing Emergency Department Walkout Rate and Boarding Hours by Improving Inpatient Length of Stay. West. J. Emerg. Med..

[88] Molla M., Warren D.S., Stewart S.L., Stocking J., Johl H., Sinigayan V. (2018). A Lean Six Sigma Quality Improvement Project Improves Timeliness of Discharge from the Hospital. Jt. Comm. J. Qual. Patient Saf..

[89] King D.L., Ben-Tovim D.I., Bassham J. (2006). Redesigning emergency department patient flows: Application of Lean Thinking to health care. Emerg. Med. Australas..

[90] Recht M., Block K.T., Chandarana H., Friedland J., Mullholland T., Teahan D., Wiggins R. (2019). Optimization of MRI turnaround times through the use of dockable tables and innovative architectural design strategies. Am. J. Roentgenol..

[91] Castaldi M., Sugano D., Kreps K., Cassidy A., Kaban J. (2016). Lean philosophy and the public hospital. Perioper. Care Oper. Room Manag..

[92] Hassanain M., Zamakhshary M., Farhat G., Al-Badr A. (2017). Use of Lean methodology to improve operating room efficiency in hospitals across the Kingdom of Saudi Arabia. Int. J. Health Plan. Manag..

[93] Bender J., Nicolescu T., Hollingsworth S.B., Murer K., Wallace K.R., Ertl W.J. (2015). Improving operating room efficiency via an interprofessional approach. Am. J. Surg..

[94] Zepeda-Lugo C., Tlapa D., Baez-Lopez Y., Limon-Romero J., Ontiveros S., Perez-Sanchez A., Tortorella G. (2020). Assessing the impact of lean healthcare on inpatient care: A systematic review. Int. J. Environ. Res. Public Health.

[95] Nicosia F.M., Park L.G., Gray C.P., Yakir M.J., Hung D.Y. (2018). Nurses’ Perspectives on Lean Redesigns to Patient Flow and Inpatient Discharge Process Efficiency. Glob. Qual. Nurs. Res..

[96] Moher D., Liberati A., Tetzlaff J., Altman D.G., The PRISMA Group (2009). Preferred Reporting Items for Systematic Reviews and Meta-Analyses: The PRISMA Statement. PLoS Med..

[97] Liberati A., Altman D.G., Tetzlaff J., Mulrow C., Gøtzsche P.C., Ioannidis J.P.A.A., Clarke M., Devereaux P.J.J., Kleijnen J., Moher D. (2009). The PRISMA statement for reporting systematic reviews and meta-analyses of studies that evaluate health care interventions: Explanation and elaboration. PLoS Med..

[98] Page M.J., McKenzie J.E., Bossuyt P.M., Boutron I., Hoffmann T.C., Mulrow C.D., Shamseer L., Tetzlaff J.M., Akl E.A., Brennan S.E. (2021). The PRISMA 2020 statement: An updated guideline for reporting systematic reviews. BMJ.

[99] Higgins J., Green S., Higgins J., Green S. (2011). Cochrane Handbook for Systematic Reviews of Interventions.

[100] McGowan J., Sampson M., Salzwedel D.M., Cogo E., Foerster V., Lefebvre C. (2016). PRESS Peer Review of Electronic Search Strategies: 2015 Guideline Statement. J. Clin. Epidemiol..

[101] Tlapa D., Zepeda-Lugo C.A., Tortorella G.L., Baez-Lopez Y.A., Limon-Romero J., Alvarado-Iniesta A., Rodriguez-Borbon M.I. (2020). Effects of Lean Healthcare on Patient Flow: A Systematic Review. Value Health.

[102] Moraros J., Lemstra M., Nwankwo C. (2016). Lean interventions in healthcare: Do they actually work? A systematic literature review. Int. J. Qual. Health Care.

[103] Centers for Medicare & Medicaid Services HCAHPS Survey. http://www.hcahpsonline.org/files/2017_SurveyInstruments_English_Mail.pdf.

[104] Sterne J.A.C., Higgins J.P.T., Elbers R.G., Reeves B.C., The Development Group for ROBINS-I Risk of Bias in Non-randomized Studies of Interventions (ROBINS-I): Detailed Guidance, Updated 12 October 2016. http://www.riskofbias.info.

[105] Sterne J., Hernán M., Reeves B., Savović J., Berkman N.D., Viswanathan M., Henry D., Altman D., Ansari M., Boutron I. (2016). ROBINS-I: A tool for assessing risk of bias in non-randomised studies of interventions. BMJ.

[106] Shortell S., Blodgett J., Rundall T., Kralovec P. (2018). Use of Lean and Related Transformational Performance Improvement Systems in Hospitals in the United States: Results From a National Survey. Jt. Comm. J. Qual. Patient Saf..

[107] Lo M., Rutman L., Migita R., Woodward G. (2015). Rapid electronic provider documentation design and implementation in an academic pediatric emergency department. Pediatr. Emerg. Care.

[108] Beck M., Gosik K. (2015). Redesigning an inpatient pediatric service using Lean to improve throughput efficiency. J. Hosp. Med..

[109] Baril C., Gascon V., Miller J., Côté N. (2016). Use of a discrete-event simulation in a Kaizen event: A case study in healthcare. Eur. J. Oper. Res..

[110] Ortiz-Barrios M., Alfaro-Saiz J.J. (2020). An integrated approach for designing in-time and economically sustainable emergency care networks: A case study in the public sector. PLoS ONE.

[111] Shilpasree A., Chandra P., Patil V., Kulkarni S., Muddaraddi R., Patil V., Ingleshwar D. (2019). Effectiveness of Implementing Process Improvement Strategies on Turnaround Time of Emergency Investigations, in Clinical Biochemistry Laboratory. Indian J. Med. Biochem..

[112] Thureson J. (2015). Reducing the Turnaround time in the Histopathology Service—Experiences of an Improvement Process. http://www.diva-portal.org/smash/get/diva2:821107/FULLTEXT01.pdf.

[113] Jensen K., Haniff R., Kamarinos A., Rosenberg A., Santiago M., Laser J. (2019). Improving Turnaround Times through a Process Improvement Initiative Involving Barcoding, Floorplans, Dual Measuring Cells, Chemistry Analyzers, and Staff Shifts. J. Appl. Lab. Med..

[114] Bhat S., Gijo E.V., Jnanesh N.A. (2016). Productivity and performance improvement in the medical records department of a hospital An application of Lean Six Sigma. Int. J. Product. Perform. Manag..

[115] Sanders J., Karr T. (2015). Improving ED specimen TAT using Lean Six Sigma. Int. J. Health Care Qual. Assur..

[116] Wannemuehler T.J., Elghouche A.N., Kokoska M.S., Deig C.R., Matt B.H. (2015). Impact of Lean on surgical instrument reduction: Less is more. Laryngoscope.

[117] White B., Chang Y., Grabowski B., Brown D. (2014). Using lean-based systems engineering to increase capacity in the emergency department. West. J. Emerg. Med..

[118] Nelson-Peterson D.L., Leppa C.J. (2007). Creating an environment for caring using lean principles of the Virginia Mason production system. J. Nurs. Adm..

[119] Furterer S.L. (2014). Hospital and Emergency Department Throughput Improvement. Lean Six Sigma Case Studies in the Healthcare Enterprise.

[120] Tsai H.W., Huang S.W., Hung Y.L., Hsu Y.S., Huang C.C. (2021). Use of the smart lean method to conduct high-quality integrated perioperative management prior to hospitalization. Int. J. Environ. Res. Public Health.

[121] Rutman L., Stone K., Reid J., Woodward G.A.T., Migita R. (2015). Improving patient flow using lean methodology: An emergency medicine experience. Curr. Treat. Options Pediatr..

[122] Tejedor-Panchón F., Montero-Pérez F.J., Tejedor-Fernández M., Jiménez-Murillo L., Calderón De La Barca-Gázquez J.M., Quero-Espinosa F.B. (2014). Improvement in hospital emergency department processes with application of lean methods. Emergencias.

[123] Eller A. (2009). Rapid assessment and disposition: Applying LEAN in the emergency department. J. Healthc. Qual..

[124] Rico F., Yalcin A., Eikman E.A. (2015). Technology Integration Performance Assessment Using Lean Principles in Health Care. Am. J. Med. Qual..

[125] Garza-Reyes J., Villarreal B., Kumar V., Diaz-Ramirez J. (2019). A lean-TOC approach for improving Emergency Medical Services (EMS) transport and logistics operations. Int. J. Logist. Res. Appl..

[126] Amati M., Valnegri A., Bressan A., La Regina D., Tassone C., Lo Piccolo A., Mongelli F., Saporito A. (2022). Reducing Changeover Time Between Surgeries Through Lean Thinking: An Action Research Project. Front. Med..

[127] Baslyman M., Amyot D., Alshalahi Y. (2019). Lean healthcare processes: Effective technology integration and comprehensive decision support using requirements engineering methods. Proceedings of the 2019 IEEE/ACM 1st International Workshop on Software Engineering for Healthcare (SHE).

[128] Aggarwal A., Aeran H., Rathee M. (2019). Quality management in healthcare: The pivotal desideratum. J. Oral Biol. Craniofacial Res..

[129] Poksinska B.B., Fialkowska-Filipek M., Engström J. (2017). Does Lean healthcare improve patient satisfaction? A mixed-method investigation into primary care. BMJ Qual. Saf..

[130] Yang T., Wang T.K., Li V.C., Su C.L. (2015). The Optimization of Total Laboratory Automation by Simulation of a Pull-Strategy. J. Med. Syst..

[131] Kane M., Chui K., Rimicci J., Callagy P., Hereford J., Shen S., Norris R., Pickham D. (2015). Lean manufacturing improves emergency department throughput and patient satisfaction. J. Nurs. Adm..

[132] Sampalli T., Desy M., Dhir M., Edwards L., Dickson R., Blackmore G. (2015). Improving wait times to care for individuals with multimorbidities and complex conditions using value stream mapping. Int. J. Health Policy Manag..

[133] Singer S.J. (2022). Value of a value culture survey for improving healthcare quality. BMJ Qual. Saf..

[134] Zegers M., Veenstra G.L., Gerritsen G., Verhage R., van der Hoeven H.J.G., Welker G.A. (2022). Perceived Burden Due to Registrations for Quality Monitoring and Improvement in Hospitals: A Mixed Methods Study. Int. J. Health Policy Manag..

[135] Taylor S., McSherry R., Cook S., Giles E. (2021). Exploring the emotional experience of lean. J. Health Organ. Manag..

[136] Po J., Rundall T.G., Shortell S.M., Blodgett J.C. (2019). Lean Management and U.S. Public Hospital Performance: Results from a National Survey. J. Healthc. Manag..

[137] Tortorella G.L., Fogliatto F.S., Espôsto K.F., Mac Cawley A.F., Vassolo R., Tlapa D., Narayanamurthy G. (2022). Healthcare costs’ reduction through the integration of Healthcare 4.0 technologies in developing economies. Total Qual. Manag. Bus. Excell..

[138] Vassolo R., Mac Cawley A., Tortorella G.L., Fogliatto F.S., Tlapa Mendoza D., Narayanamurthy G. (2021). Hospital Investments Decisions in Healthcare 4.0 Technologies: Challenges, Trends, and Research Directions. J. Med. Internet Res..

[139] Mourtzis D. (2020). Simulation in the design and operation of manufacturing systems: State of the art and new trends. Int. J. Prod. Res..

[140] Ortíz-Barrios M., Escorcia-Caballero J., Sánchez-Sánchez F., De Felice F., Petrillo A. (2017). Efficiency Analysis of Integrated Public Hospital Networks in Outpatient Internal Medicine. J. Med. Syst..

[141] Noto G., Cosenz F. (2020). Introducing a strategic perspective in lean thinking applications through system dynamics modelling: The dynamic Value Stream Map. Bus. Process Manag. J..

[142] Lokesh K., Samanta A.K., Varaprasad G. (2020). Reducing the turnaround time of laboratory samples by using Lean Six Sigma methodology in a tertiary-care hospital in India. Proceedings of the 2020 International Conference on System, Computation, Automation and Networking (ICSCAN).

[143] Bhosekar A., Ekşioğlu S., Işık T., Allen R. (2021). A discrete event simulation model for coordinating inventory management and material handling in hospitals. Ann. Oper. Res..

[144] Romano E., Falegnami A., Cagliano A.C., Rafele C. (2022). Lean ICU Layout Re-Design: A Simulation-Based Approach. Informatics.

[145] Hirisatja T., Lila B., Chantrasa R. Healthcare Operations Improvement with an Integration of Discrete-Event Simulation and Lean Thinking. Proceedings of the International conference on Innovative Engineering Technologies.

[146] Converso G., Improta G., Mignano M., Santillo L.C. (2015). A Simulation approach for Implementing of Agile Production Logic for a Hospital Emergency Unit, Intell. Softw. Method. Tools Tech.

[147] Lin W.D., Jin X., Chia S.Y. Simulation based lean six sigma approach to reduce patients waiting time in an outpatient eye clinic. Proceedings of the 2014 IEEE International Conference on Industrial Engineering and Engineering Management.

[148] Haddad M., Zouein P., Salem J., Otayek R. (2016). Case Study of Lean in Hospital Admissions to Inspire Culture Change. EMJ Eng. Manag. J..

[149] Doğan N.Ö., Unutulmaz O. (2016). Lean production in healthcare: A simulation-based value stream mapping in the physical therapy and rehabilitation department of a public hospital. Total Qual. Manag. Bus. Excell..

[150] Salam M.A., Khan S.A. (2016). Value creation through lean management: A case study of healthcare service operations. Int. J. Serv. Oper. Manag..

[151] Al-Zain Y., Al-Fandi L., Arafeh M., Salim S., Al-Quraini S., Al-Yaseen A., Abu Taleb D. (2019). Implementing Lean Six Sigma in a Kuwaiti private hospital. Int. J. Health Care Qual. Assur..

[152] Agnetis A., Bianciardi C., Iasparra N. (2019). Integrating lean thinking and mathematical optimization: A case study in appointment scheduling of hematological treatments. Oper. Res. Perspect..

[153] Tortorella G., Sawhney R., Jurburg D., de Paula I.C., Tlapa D., Thurer M. (2020). Towards the proposition of a Lean Automation framework. J. Manuf. Technol. Manag..

[154] Senna P., Reis A., Dias A., Coelho O., Guimarães J., Eliana S. (2021). Healthcare supply chain resilience framework: Antecedents, mediators, consequents. Prod. Plan. Control.

[155] Rosas-Hernandez L., Tlapa D., Baez-Lopez Y., Limon-Romero J., Perez-Sanchez A. (2021). Lean Healthcare y DMAIC para mejorar el proceso de suministro en un hospital público. DYNA Manag..

[156] Sheehan J.R., Lyons B., Holt F. (2021). The use of Lean Methodology to reduce personal protective equipment wastage in children undergoing congenital cardiac surgery, during the COVID-19 pandemic. Paediatr. Anaesth..

[157] Noronha A., Bhat S., Gijo E.V., Antony J., Bhat S. (2022). Application of Lean Six Sigma in conservative dentistry: An action research at an Indian dental college. TQM J..

[158] Martin L., Lyons M., Patton A., O Driscoll M., McLoughlin K., Hannon E., Deasy C. (2022). Implementing delirium screening in the emergency department: A quality improvement project. BMJ Open Qual..

[159] Gunasekeran D.V., Tseng R.M.W.W., Tham Y.C., Wong T.Y. (2021). Applications of digital health for public health responses to COVID-19: A systematic scoping review of artificial intelligence, telehealth and related technologies. NPJ Digit. Med..

[160] Ilangakoon T.S., Weerabahu S.K., Samaranayake P., Wickramarachchi R. (2022). Adoption of Industry 4.0 and lean concepts in hospitals for healthcare operational performance improvement. Int. J. Prod. Perform. Manag..

[161] Rosen R., Von Wichert G., Lo G., Bettenhausen K.D. (2015). About the importance of autonomy and digital twins for the future of manufacturing. IFAC-PapersOnLine.

[162] Rodič B. (2017). Industry 4.0 and the New Simulation Modelling Paradigm. Organizacija.

[163] Hoot N., Aronsky D. (2008). Systematic Review of Emergency Department Crowding: Causes, Effects, and Solutions. Ann. Emerg. Med..

[164] Cochran D., Swartz J., Elahi B., Smith J. (2018). Using the Collective System Design Methodology to Improve a Medical Center Emergency Room Performance. J. Med. Syst..

[165] Marques da Rosa V., Saurin T.A., Tortorella G.L., Fogliatto F.S., Tonetto L.M., Samson D. (2021). Digital technologies: An exploratory study of their role in the resilience of healthcare services. Appl. Ergon..

[166] Mohamed N., Al-Jaroodi J. The impact of industry 4.0 on healthcare system engineering. Proceedings of the SysCon 2019—13th Annual IEEE International Systems Conference.

[167] Rundall T., Shortell S., Blodgett J., Henke R.M., Foster D. (2021). Adoption of Lean management and hospital performance: Results from a national survey. Health Care Manag. Rev..

[168] Bergey M., Goldsack J., Robinson E. (2019). Invisible work and changing roles: Health information technology implementation and reorganization of work practices for the inpatient nursing team. Soc. Sci. Med..

[169] Fournier P.L., Jobin M.H., Lapointe L., Bahl L. (2021). Lean implementation in healthcare: Offsetting Physicians’ resistance to change. Prod. Plan. Control.

[170] Leite H., Williams S., Radnor Z., Bateman N. (2022). Emergent barriers to the lean healthcare journey: Baronies, tribalism and scepticism. Prod. Plan. Control.

[171] Akmal A., Foote J., Podgorodnichenko N., Greatbanks R., Gauld R. (2022). Understanding resistance in lean implementation in healthcare environments: An institutional logics perspective. Prod. Plan. Control.

[172] Harrison M., Paez K., Carman K., Stephens J., Smeeding L., Devers K., Garfinkel S. (2016). Effects of organizational context on Lean implementation in five hospital systems. Health Care Manag. Rev..

[173] Chen P.S., Yu C.J., Chen G.Y.H. (2015). Applying Task-Technology Fit Model to the Healthcare Sector: A Case Study of Hospitals’ Computed Tomography Patient-Referral Mechanism. J. Med. Syst..

[174] Pennathur P.R., Bisantz A.M., Fairbanks R.J., Perry S.J., Zwemer F., Wears R.L. (2007). Assessing the impact of computerization on work practice: Information technology in emergency departments. Proceedings of the Human Factors and Ergonomics Society.

[175] Dos Santos B.M., Fogliatto F.S., Zani C.M., Peres F.A.P. (2021). Approaches to the rationalization of surgical instrument trays: Scoping review and research agenda. BMC Health Serv. Res..

[176] Chua W.L., See M.T.A., Legio-Quigley H., Jones D., Tee A., Liaw S.Y. (2017). Factors influencing the activation of the rapid response system for clinically deteriorating patients by frontline ward clinicians: A systematic review. Int. J. Qual. Health Care.

[177] Beaudry A., Laporte G., Melo T., Nickel S. (2010). Dynamic transportation of patients in hospitals. OR Spectr..

[178] Chiarini A. (2013). Waste savings in patient transportation inside large hospitals using lean thinking tools and logistic solutions. Leadersh. Health Serv..

[179] Peimbert-García R.E., Gutiérrez-Mendoza L.M., García-Reyes H. (2021). Applying lean healthcare to improve the discharge process in a mexican academic medical center. Sustainability.

[180] Nagi A., Altarazi S. (2017). Integration of value stream map and strategic layout planning into DMAIC approach to improve carpeting process. J. Ind. Eng. Manag..

[181] Fogliatto F.S., Tortorella G.L., Anzanello M.J., Tonetto L.M. (2019). Lean-oriented layout design of a health care facility. Qual. Manag. Health Care.

[182] Li Z.H., Zhong Y.F. (2003). Virtual Facility Layout Design Using Virtual Reality Techniques. Wuhan Univ. J. Nat. Sci. A.

