# Supplementary material for: Effects of Lean Interventions Supported by Digital Technologies on Healthcare Services: A Systematic Review
Source: Int J Environ Res Public Health. 2022 Jul 25;19(15):9018. doi: 10.3390/ijerph19159018 (PMC9330917; doi:10.3390/ijerph19159018)
Supplement: Supplementary file 1 [file ijerph-19-09018-s001.zip › ijerph-1809987-supplementary.pdf]

Table S1. PRISMA Checklist

Section and Topic	Item #	Checklist item	Location where item is reported
**TITLE**			
Title	1	Identify the report as a systematic review.	Title
**ABSTRACT**			
Abstract	2	See the PRISMA 2020 for Abstracts checklist (Background, Method, Results, and Discussion)	Abstract
**INTRODUCTION**			
Rationale	3	Describe the rationale for the review in the context of existing knowledge.	Introduction
Objectives	4	Provide an explicit statement of the objective(s) or question(s) the review addresses.	Introduction
**METHODS**			
Eligibility criteria	5	Specify the inclusion and exclusion criteria for the review and how studies were grouped for the syntheses.	Methods
Information sources	6	Specify all databases, registers, websites, organisations, reference lists and other sources searched or consulted to identify studies. Specify the date when each source was last searched or consulted.	Methods
Search strategy	7	Present the full search strategies for all databases, registers and websites, including any filters and limits used.	Table S2
Selection process	8	Specify the methods used to decide whether a study met the inclusion criteria of the review, including how many reviewers screened each record and each report retrieved, whether they worked independently, and if applicable, details of automation tools used in the process.	Methods
Data collection process	9	Specify the methods used to collect data from reports, including how many reviewers collected data from each report, whether they worked independently, any processes for obtaining or confirming data from study investigators, and if applicable, details of automation tools used in the process.	Methods
Data items	10a	List and define all outcomes for which data were sought. Specify whether all results that were compatible with each outcome domain in each study were sought (e.g. for all measures, time points, analyses), and if not, the methods used to decide which results to collect.	Methods
	10b	List and define all other variables for which data were sought (e.g. participant and intervention characteristics, funding sources). Describe any assumptions made about any missing or unclear information.	Methods
Study risk of bias assessment	11	Specify the methods used to assess risk of bias in the included studies, including details of the tool(s) used, how many reviewers assessed each study and whether they worked independently, and if applicable, details of automation tools used in the process.	Methods
Effect measures	12	Specify for each outcome the effect measure(s) (e.g. risk ratio, mean difference) used in the synthesis or presentation of results.	Methods and Results
Synthesis methods	13a	Describe the processes used to decide which studies were eligible for each synthesis (e.g. tabulating the study intervention characteristics and comparing against the planned groups for each synthesis (item #5)).	Methods
	13b	Describe any methods required to prepare the data for presentation or synthesis, such as handling of missing summary statistics, or data conversions.	Methods

Section and Topic	Item #	Checklist item	Location where item is reported
	13c	Describe any methods used to tabulate or visually display results of individual studies and syntheses.	Methods
	13d	Describe any methods used to synthesize results and provide a rationale for the choice(s). If meta-analysis was performed, describe the model(s), method(s) to identify the presence and extent of statistical heterogeneity, and software package(s) used.	Methods
	13e	Describe any methods used to explore possible causes of heterogeneity among study results (e.g. subgroup analysis, meta-regression).	Not applicable
	13f	Describe any sensitivity analyses conducted to assess robustness of the synthesized results.	Not applicable
Reporting bias assessment	14	Describe any methods used to assess risk of bias due to missing results in a synthesis (arising from reporting biases).	Methods
Certainty assessment	15	Describe any methods used to assess certainty (or confidence) in the body of evidence for an outcome.	Not applicable
**RESULTS**			
Study selection	16a	Describe the results of the search and selection process, from the number of records identified in the search to the number of studies included in the review, ideally using a flow diagram.	Results
	16b	Cite studies that might appear to meet the inclusion criteria, but which were excluded, and explain why they were excluded.	Results and Discussion
Study characteristics	17	Cite each included study and present its characteristics.	Results
Risk of bias in studies	18	Present assessments of risk of bias for each included study.	Table S4
Results of individual studies	19	For all outcomes, present, for each study: (a) summary statistics for each group (where appropriate) and (b) an effect estimate and its precision (e.g. confidence/credible interval), ideally using structured tables or plots.	Tables 1-5
Results of syntheses	20a	For each synthesis, briefly summarise the characteristics and risk of bias among contributing studies.	Results
	20b	Present results of all statistical syntheses conducted. If meta-analysis was done, present for each the summary estimate and its precision (e.g. confidence/credible interval) and measures of statistical heterogeneity. If comparing groups, describe the direction of the effect.	Not applicable
	20c	Present results of all investigations of possible causes of heterogeneity among study results.	Discussion
	20d	Present results of all sensitivity analyses conducted to assess the robustness of the synthesized results.	Not applicable
Reporting biases	21	Present assessments of risk of bias due to missing results (arising from reporting biases) for each synthesis assessed.	Not applicable
Certainty of evidence	22	Present assessments of certainty (or confidence) in the body of evidence for each outcome assessed.	Not applicable
**DISCUSSION**			
Discussion	23a	Provide a general interpretation of the results in the context of other evidence.	Discussion
	23b	Discuss any limitations of the evidence included in the review.	Discussion
	23c	Discuss any limitations of the review processes used.	Discussion
	23d	Discuss implications of the results for practice, policy, and future research.	Discussion

Section and Topic	Item #	Checklist item	Location where item is reported
**OTHER INFORMATION**			
Registration and protocol	24a	Provide registration information for the review, including register name and registration number, or state that the review was not registered.	Methods
	24b	Indicate where the review protocol can be accessed, or state that a protocol was not prepared.	Methods
	24c	Describe and explain any amendments to information provided at registration or in the protocol.	Not applicable
Support	25	Describe sources of financial or non-financial support for the review, and the role of the funders or sponsors in the review.	Not applicable
Competing interests	26	Declare any competing interests of review authors.	Conclusions
Availability of data, code and other materials	27	Report which of the following are publicly available and where they can be found: template data collection forms; data extracted from included studies; data used for all analyses; analytic code; any other materials used in the review.	Table S3

From: Page MJ, McKenzie JE, Bossuyt PM, Boutron I, Hoffmann TC, Mulrow CD, et al. The PRISMA 2020 statement: an updated guideline for reporting systematic reviews. BMJ 2021;372:n71. doi: 10.1136/bmj.n71

For more information, visit: http://www.prisma-statement.org/

Table S2. Search Strategy

PubMed-Medline

(Total Quality Management[MH] OR Lean Healthcare[TW] OR Lean Method*[TW] OR Lean Philosophy[TW] OR Lean Management[TW] OR Lean six sigma*[TW] OR Toyota Production System*[TW] OR Six sigma) AND (Information technolog*[TW] OR Communication technolog*[TW] OR Healthcare technolog*[TW] OR Healthcare 4.0[TW] OR H4.0[TW] OR Ehealth[TW] OR Health information system*[TW] OR Smart Technolog*[TW] OR Telemedicine[TW] OR Internet of things[TW] OR Big Data[TW] OR Cloud computing[TW] OR Fog computing[TW] OR Mobile Computing[TW] OR Remote Sens*[TW] OR Robot*[TW] OR Artificial intelligence[TW] OR 3D print*[TW] OR Virtual reality[TW] OR Augmented reality[TW] OR Automat*[TW] OR Simulat*[TW] OR Open source software[TW] OR Blockchain[TW] OR Electronic medical record*[TW] OR Electronic health record*[TW]) AND (Length of Stay[TW] OR LOS OR Turnover Time[TW] OR TOT OR Turnaround Time[TW] OR TAT OR Boarding Time[TW] OR Wait* Time[TW] OR Patient Left Without Being Seen[TW] OR LWBS)

Cochrane Library-CENTRAL

1	[mh Total Quality Management]
2	(Lean near Healthcare) OR (Lean near Method*) OR (Lean near Philosophy) OR (Lean near Management) OR (Lean near six sigma*) OR (Toyota Production System*) OR (Six near sigma)
3	#1 OR #2
4	(Information near technolog*) OR (Communication near technolog*) OR (Healthcare near technolog*) OR (Healthcare near 4.0) OR (H4.0) OR (Ehealth) OR (Health near information near system*) OR (Smart near Technolog*) OR (Telemedicine) OR (Internet near of near thing*) OR (Big near Data) OR (Cloud near computing) OR (Fog near computing) OR (Mobile near Computing) OR (Remote near Sens*) OR (Robot*) OR (Artificial near intelligence) OR (3D near print*) OR (Virtual near reality) OR (Augmented near reality) OR (Automat*) OR (Simulat*) OR (Open near source near software) OR (Blockchain) OR (Electronic near medical near record*) OR (Electronic near health near record*)
5	(Length near Stay) OR (LOS) OR (Turnover near Time) OR (TOT) OR (Turnaround near time) OR (TAT) OR (Boarding near Time) OR (Wait* near Time) OR (Patient near Left near Without near Being near Seen) OR (LWBS)
6	{AND #3-#5}

Ebsco-Host / CINAHL

Total Quality Management OR Lean Healthcare OR Lean Method* OR Lean Philosophy OR Lean Management OR Lean six sigma* OR Toyota Production System* OR Six sigma

AND

Information technolog* OR Communication technolog* OR Healthcare technolog* OR Healthcare 4.0 OR H4.0 OR Ehealth OR Health information system* OR Smart Technolog* OR Telemedicine OR Internet of things OR Big Data OR Cloud computing OR Fog computing OR Mobile Computing OR Remote Sens* OR Robot* OR Artificial intelligence OR 3D print* OR Virtual reality OR Augmented reality OR Automat* OR Simulat* OR Open source software OR Blockchain OR Electronic medical record* OR Electronic health record*

AND

Length of Stay OR LOS OR Turnover Time OR TOT OR Turnaround Time OR TAT OR Boarding Time OR Wait* Time OR Patient Left Without Being Seen OR LWBS

AND

Hospital OR Healthcare OR Health care OR Clinic* OR Health OR Medic*

AND

Lean Six Sigma

Web of Science

Total Quality Management OR Lean Healthcare OR Lean Method* OR Lean Philosophy OR Lean Management OR Lean six sigma* OR Toyota Production System* OR Six sigma

AND

Information technolog* OR Communication technolog* OR Healthcare technolog* OR Healthcare 4.0 OR H4.0 OR Ehealth OR Health information system* OR Smart Technolog* OR Telemedicine OR Internet of things OR Big Data OR Cloud computing OR Fog computing OR Mobile Computing OR Remote Sens* OR Robot* OR Artificial intelligence OR 3D print* OR Virtual reality OR Augmented reality OR Automat* OR Simulat* OR Open source software OR Blockchain OR Electronic medical record* OR Electronic health record*

AND

Length of Stay OR LOS OR Turnover Time OR TOT OR Turnaround Time OR TAT OR Boarding Time OR Wait* Time OR Patient Left Without Being Seen OR LWBS

AND

Hospital OR Healthcare OR Health care OR Clinic* OR Health OR Medic*

Table S3. Extended summary of findings

First Author, Year, Country	Setting; Study Design; n; Time Frame	Main Intervention	Outcomes	Summary of findings
(Amati et al., 2022) Switzerland	Operating room; Case study, pre-post; 9 months	Lean and simulation	Mean changeover time for gynecological surgery (skin-to skin)	Reduced from 58 min to 41 min
			Mean changeover time for general surgery (skin-to skin)	Reduced from 63 min to 48 min
			Potential savings	1500 CHF per room per day
Tsai et al., 2021)Taiwan	Operating room; Case study, pre-post; n=2,964; 24 months	Lean six sigma, electronic tracking system including electronic tags, registration APP for positioning and directions, QR-codes, perioperative flow system, and Health Information System	Mean LOS (orthopedy surgery)	Reduced from 3.31 days to 1.57 days
			Mean LOS (colon and rectal surgery)	Reduced from 2.49 days to 1.16 days
			Mean LOS (urology surgery)	Reduced from 3.31 days to 1.57 days
			Mean LOS (otorhinolaryngolog y division	Reduced from 2.49 days to 1.16 days
			Hospitalization cycle time	Reduced from 96 min to 73 min
			Mean Non-Value-Added time	Reduced from 62 min to 42 min
			Percentage of Process Cycle Efficiency	Increased from 35.42% to 42.47%
			Mean Patient satisfaction	Increased from 4.29 to 4.40 (p<0.05)
(Wongkrajang et al., 2020) Thailand	Laboratory; Case study, pre-post; n=30,180; 3 months	Lean and automation	90th percentile Turnaround time	Reduced from 60 min to 50 min (p=0.01)
(Ortiz-Barrios & Alfaro-Saiz, 2020) Colombia	ED; case study; n= 16,741; 15 months	Lean, simulation and virtual modelling	Mean waiting time	Reduced from 201.6 min to 103.1 min
(Ankrum et al., 2019) USA	Pediatric facility; case study, pre-post; n=47 room turnovers; 60 days	Lean, robotics, and electronic medical records	Median room turnover time	Reduced from 130 min to 65 min (p< 0.0001)
			Median time between room breakdown to cleaning start time	Reduced from 10 min to 3 min (p< 0.004)
(Recht et al., 2019) USA	MRI; case study, control vs intervention; n1=5,461 and n2=9,221; 6 months	Lean and automation of MRI (software tool)	Mean turnaround time (patients ready for scanning)	Reduced from 328 min to 132 min (p<0.0001)
			Mean turnaround time (all patients assessed)	Reduced from 537 min to 272 min (p<0.0001)

(Baslyman et al., 2019) Saudi Arabia and Canada	Emergency room and lab; intervention (RTTS) vs control	Lean, real-time tracking sample or RTTS (RFID), and automated notification system	Patient satisfaction	Current=227 vs RTTS=663
			Emergency room satisfaction	Current=25 vs RTTS=242
			Lab satisfaction	Current=0 vs RTTS=300
(Shilpasree et al., 2019) India	Clinical laboratory; pre-post; n= 3,344; 2 months	Lean six sigma, automation, and computerization	Turnaround time	Reduced from 110 min to 78.7 min ($p<0.001$)
(Jensen et al., 2019) USA	Laboratory; pre-post; n= 21,639; 20 months; 4 months follow up	Lean and automation (automated chemistry line and barcoding)	Specimen turnaround time	TnT: reduced from 56.64 min to 53.68 min ($p< 0.001$)
				K+: reduced from 40.88 min to 39.82 min ($p<0.001$)
				CMP-Alb: reduced from 43.44 min to 40.51 min ($p< 0.001$)
(Garza-Reyes et al., 2019) Mexico	Ambulance service; case study; n=850 ambulances; 1 month	Lean and simulation, internet-based technologies, and GPS tracking devices	Average ambulance cycle time	Reduced from 124.9 min to 75.8 min
(Brunsman, 2018) USA	Inpatient pharmacy, cohort study, (n=102), 15 months	Lean and automation of dispensing cabinet	Median length of stay	Reduced from 22.9 days to 13.2 days ($p=0.049$)
			Median time from order to medication verification	Increased from 5.5 min to 10.5 min ($p=0.11$)
			Median overall turnaround time from CMS-approved antibiotic order entry to medication administration.	Reduced from 120 min to 80 min ($p=0.014$)
(Baril et al., 2016) Canada	Hematology–oncology clinic; case study; 10 months, 2 months of follow up	Lean, simulation and business game-virtual environment	Mean patient waiting time before treatment	Reduced from 61 min to 16 min
(Bhat et al., 2016) India	Medical record department; case study; n=100; 2 months	Lean six sigma and simulation	Turnaround time of medical records preparation	Reduced from 19 min to 8 min
			Potential savings	US $20,000 annually
(Rutman et al., 2015) USA	ED; pre-post; n=98; 7 months	Lean, simulation and electronic medical records	Median time to see a provider	Reduced from 43 min to 7 min
			Patients seen within 30 min	Increased from 33 to 93%
			Mean LOS in ED	Reduced by 30 min
(Bender et al., 2015) USA	Operating room; pre-post; n=25,903; 36 months	Lean six sigma and robots	On-time starts	Increased from 32 to 73%
			Mean turnover time	Non-significant change from 43 min to 44 min

			Actual room utilization	Improved from 56% to 68%
			Percentage of overtime	Reduced from 7% to 4 %
(Beck & Gosik, 2015) USA	Inpatient pediatric service; pre-post; n=3,509; 12 months	Lean six sigma and tele-tracking systems	Median time of patient discharge	Reduced from 15:48 to 14:1 (p<0.0001)
			Percentage of patients discharged by noon	Increased from 14% to 27% (p<0.0001)
			Mean length of stay	Non-significant change, from 3.1 days to 3.0 days (p=0.864)
			30-day readmission rates	Non-significant change, from 9.4% to 9.1% (p=0.703)
(Lee et al., 2015) USA	Emergency care center; n=18,726; 9 months	Process mapping, machine learning, simulation, and optimization	Overall LOS	Reduced from 10.59 h to 7.14 h
			Percentage of patients LWBS	Reduced by 30%
			Percentage of 30-day Readmission rate	Reduced by 30%
			ED costs reductions and savings in penalties (from 2008-2012)	US $29.1 million
(Rico et al., 2015) USA	ED; pre-post; n=50; 1 month	Lean and automated infusion system	Mean waiting time for FDG Infusion	Reduced from 11.3 min to 6.4 min (p<0.01)
(Thureson, 2015) Sweden	Histopathology lab; pre-post; n=46,675; 27 months	Lean and automatic embedding console (digital pathology)	Median TAT for patients with breast cancer	Reduced from 25 days to 15.5 days (p<0.001)
(Lo et al., 2015) USA	Pediatric emergency department; pre-post; 7 months	Lean, real-time voice recognition system, simulation, electronic charting, and EHR	Discharged patients LOS	Increased from 161 min to 168 min
			Admitted patients LOS	No changed (270 min)
(Sanders & Karr, 2015) USA	ED; Pre-post	Lean six sigma and ED tracking boards	Median TAT for ED specimens of complete blood count analysis (CBCA)	Reduced from 15 min to 11 min
(Wannemuehler et al., 2015) USA	OR; pre-post; n=644; 10 months	Lean six sigma and electronic tracking system	Number of instruments for adenotonsillectomy sets	Reduced from 52 to 24 instruments
			Median assembly times (instrument set)	Reduced from 8.4 min to 4.7 min (p<0.0001)
			Mean Mayo setup times	Reduced from 97.6 s to 76.1 s (p<0.0001)
			Mean operative times	No change from 1,773 s vs 1,631 s (p>0.05)

(Tejedor-Panchon et al., 2014) Spain	ED; Quasi-experimental pre-post study; n=256,628; 36 months	Lean, simulation and digital technology in X-ray	Mean LOS in ED (time spent in the examination area)	NUC, reduced from 80.4 min to 61.6 min (p<0.001); TC, reduced from 137.8 min to 123.8 min (p<0.05); MSC, reduced from 219.7 min to 209.3 min (p=0.108)
			Mean wait time to see a physician	Reduced from 58 min to 49.1 min (p<0.001)
			Percentage of patients LWBS	Reduced, from 2.8% to 2.0% (p<0.001)
(White et al., 2014) USA	ED; prospective controlled, pre-post study; n=59,687; 17 months.	Lean, six sigma, QT, TOC, electronic patient tracking system (EDIS)	Median LOS for discharged patients	Intervention group reduced from 158 min to 143 min (p<0.0001) No change in control group from 265 min to 267 min (p=0.69)
			Percentage of patients discharged within one hour	Intervention group increased from 6.9% to 9.7%, (p<0.0001) No change in control group from 2.9% to 2.9% (p=0.98)
			Median exam room time	Intervention group reduced 34 min from 90 to 56 min, (p< 0.0001) Control group increased from 28min to 36 min (p<0.0001)
(Furterer, 2014), USA	ED; case study; 7 months	Lean six sigma, automation, electronic ED bed board, and EMR	Mean LOS (all patients)	Reduced from 6.9 h to 4.7 h (p=0.000)
			Mean LOS (admitted patients)	Reduced from 8.7 h to 6.1 h
			Mean LOS (discharged patients)	Reduced from 5.8 h to 4.1 h
			Percentage of patients LWBS	Reduced from 6.5 % to 0.34 %
			Patient satisfaction scores	Increased by 24% to 89.9 %
			Time from door to doctor	Reduced from 100 min to 27 min
(Burkitt et al., 2009) USA	Department of surgery; cohort study; n=1,779; 48 months	TPS, automatic control of antibiotics after surgery, and computerized medical record	Median LOS	Non-significant change (p=0.90)
			Percentage of surgical admissions receiving appropriate perioperative antibiotics	Increased from 25.5% to 44% (p<0.01)

(Eller, 2009) USA	ED; pre-post; 25 months;	Lean, patient track and electronic documentation system	Mean LOS for no RAD patients	Reduction of 45 min
			Mean LOS for RAD patients	Reduction of 208 min
			Percentage of patients LWBS	Reduction of 28%
(Nelson-Peterson & Leppa, 2007) USA	General hospital; time-series, pre-post; n=8; 5 months	Lean and simulation	Staff walking distance	Reduced from 5,818 steps to 846 steps
			Registered nurse lead time	Reduced from 240 min to 126 min
			Setup time (minutes for 1 cycle of care)	Reduced from 20 min to 3 min

Note. CMP-Alb indicates complete metabolic panel-albumin; CMS, Centers for Medicare and Medicaid Services; ED, emergency department; EMR, Electronic medical records; EHR, Electronic health record; FDG, Fluorodeoxyglucose; GPS indicates global positioning system; h, hours; K+, Potassium; LOS, Length of stay; LWBS, left without being seen; MRI, Magnetic resonance imaging; MSC, medical-surgical cases; min, minutes; mo, months; NUC, non-urgent circuit; OR, Operating room; QT, Queuing theory; RAD, Rapid assessment and disposition process; TAT, Turnaround time; TC, trauma cases; TOC, Theory of constraints; TPS, Toyota production system; TnT, Troponin. The last name of the main author and the publication year are shown.

Table S4. Risk of Bias

Risk of bias domains

Study	D1	D2	D3	D4	D5	D6	D7	Overall
Nelson-Peterson 2007	-	+	+	+	+	+	-	-
Eller, 2009	-	-	+	+	+	+	-	-
Burkitt, 2009	-	+	+	+	+	-	+	-
Furterer, 2014	-	+	+	+	+	+	+	-
White, 2014	-	+	+	+	+	+	+	+
Tejedor, 2014	-	+	+	+	+	+	-	-
Wannmuehlet, 2015	-	+	-	+	+	+	+	-
Sanders, 2015	-	+	+	+	-	+	+	-
Lo, 2015	-	+	+	+	-	+	+	-
Thureson, 2015	-	+	+	+	-	+	+	-
Rico, 2015	-	+	+	+	+	-	+	-
Lee, 2015	-	+	+	+	+	-	+	-
Beck, 2015	+	+	+	+	+	+	+	+
Bender, 2015	-	+	+	+	+	-	-	X
Rutman, 20	-	-	+	+	+	+	-	-
Bhat, 2016	+	+	+	+	+	+	+	+
Baril, 2016	-	+	+	+	-	+	+	-
Brunsman, 2018	+	-	+	+	+	+	+	+
Baslyman, 2019	-	-	-	+	+	-	-	X
Garza, 2019	-	+	+	+	+	+	+	-
Jensen, 2019	-	+	+	+	+	+	+	-
Shilpasree, 2019	-	+	+	+	+	+	+	-
Recht. 2019	-	+	+	+	+	+	+	-
Ankrum, 2019	-	+	+	+	-	+	+	-
Ortiz, 2020	-	+	+	+	+	-	+	-
Wongkrajang, 2020	-	-	+	+	+	+	+	-
Tsai, 2021	-	-	+	+	+	+	+	-
Amati, 2022	-	-	+	+	+	+	+	-

Domains:
D1: Bias due to confounding.
D2: Bias due to selection of participants.
D3: Bias in classification of interventions.
D4: Bias due to deviations from intended interventions.
D5: Bias due to missing data.
D6: Bias in measurement of outcomes.
D7: Bias in selection of the reported result.

Judgement
X Serious
- Moderate
+ Low
